# Olfactory bulb neuroproteomics reveals a chronological perturbation of survival routes and a disruption of prohibitin complex during Alzheimer’s disease progression

**DOI:** 10.1038/s41598-017-09481-x

**Published:** 2017-08-22

**Authors:** Mercedes Lachén-Montes, Andrea González-Morales, María Victoria Zelaya, Estela Pérez-Valderrama, Karina Ausín, Isidro Ferrer, Joaquín Fernández-Irigoyen, Enrique Santamaría

**Affiliations:** 1 Clinical Neuroproteomics Group, Navarrabiomed, Departamento de Salud, Universidad Pública de Navarra, Pamplona, Spain; 2 IDISNA, Navarra Institute for Health Research, Pamplona, Spain; 3 Pathological Anatomy Department, Navarra Hospital Complex, Pamplona, Spain; 4 Proteored-ISCIII. Proteomics Unit, Navarrabiomed, Departamento de Salud, Universidad Pública de Navarra, Pamplona, Spain; 5 Institut de Neuropatologia, IDIBELL-Hospital Universitari de Bellvitge, Universitat de Barcelona, L’Hospitalet de Llobregat, CIBERNED (Centro de Investigación Biomédica en Red de Enfermedades Neurodegenerativas), Barcelona, Spain

## Abstract

Olfactory dysfunction is among the earliest features of Alzheimer’s disease (AD). Although neuropathological abnormalities have been detected in the olfactory bulb (OB), little is known about its dynamic biology. Here, OB- proteome analysis showed a stage-dependent synaptic proteostasis impairment during AD evolution. In addition to progressive modulation of tau and amyloid precursor protein (APP) interactomes, network-driven proteomics revealed an early disruption of upstream and downstream p38 MAPK pathway and a subsequent impairment of Phosphoinositide-dependent protein kinase 1 (PDK1)/Protein kinase C (PKC) signaling axis in the OB from AD subjects. Moreover, a mitochondrial imbalance was evidenced by a depletion of Prohibitin-2 (Phb2) levels and a specific decrease in the phosphorylated isoforms of Phb1 in intermediate and advanced AD stages. Interestingly, olfactory Phb subunits were also deregulated across different types of dementia. Phb2 showed a specific up-regulation in mixed dementia, while Phb1 isoforms were down-regulated in frontotemporal lobar degeneration (FTLD). However, no differences were observed in the olfactory expression of Phb subunits in progressive supranuclear palsy (PSP). To sum up, our data reflect, in part, the missing links in the biochemical understanding of olfactory dysfunction in AD, unveiling Phb complex as a differential driver of neurodegeneration at olfactory level.

## Introduction

Alzheimer’s disease (AD) is the most common form of senile dementia^1^. In general, two subgroups are recognized, a familial early-onset form, and a sporadic late-onset form, albeit 95% of the patients develop sporadic AD^2^. Together with typical symptoms such as memory loss and behavioral disorders, AD patients present olfactory dysfunction in 90% of the cases^3^. Interestingly, this deficit occurs at early stages of the disease and it is considered a premotor sign of neurodegeneration^3, 4^. The olfactory bulb (OB) is the first central structure of the olfactory pathway in the brain^5^. Multiple reports have evidenced neuropathological changes, and molecular alterations in the OB derived from rodent AD models, and human AD brains^6, 7^. Interestingly, the accumulation of beta-amyloid (Aβ) and phospho-Tau protein in the anterior olfactory nucleus and OB correlates with the progression of olfactory deficits and the severity of the disease in other brain regions^3^, suggesting the potential utility of olfactory tissue in the early diagnosis of AD.

Taking into account the cellular complexity and protein heterogeneity present in the OB^8, 9^, proteome-wide analysis based on high-resolution MS^10^ has become an attractive technology to characterize and quantify the OB proteome in different biological contexts^11^. Although this unbiased technology has greatly enhanced the ability to characterize novel pathways particularly in brain areas associated with AD^12, 13^, few studies have examined the proteome profiling of the early-affected OB region with the aim to investigate incipient neurodegenerative changes in AD phenotypes. Mass-spectrometric exploration of the OB derived from AD models has revealed a clear proteostasis impairment in this olfactory region. In the APP/PS1 (Amyloid precursor protein/Preselinin 1) mouse model of AD, an early dysregulation of FAK and MEK/ERK signaling pathways precedes the β-amyloid deposition in the OB^7^. Moreover, these early events are subsequently accompanied by multiple proteomic, phosphoproteomic, and glycoproteomic changes in the OB, leading to a disruption in signaling pathways related to synaptic plasticity and cytoskeletal dynamics during the progression of AD-associated amyloid pathology in APP/PS1 mice^14^. However, the AD progression in APP/PS1 mice is reminiscent of, but not identical to human sporadic AD^15^. We consider that deciphering the progressive proteome-wide alterations that occurs in a stage-dependent manner in the human OB, might complement the integrated view of the biochemical pathways involved in the olfactory pathophysiology of AD. In this study, we used a discovery platform combining neuropathological diagnosis, label-free quantitative proteomics, physical and functional interaction data, and biochemical approaches in order to understand the means by which the molecular pathways harboured in the OB are chronologically regulated during AD progression. We have revealed an olfactory proteostasis impairment across neuropathological grading detecting: i) differential expression of 278 proteins between controls and AD phenotypes, ii) a progressive modulation of APP, and Tau interactome networks across AD stages, iii) alteration in MKK3-6/p38 MAPK, and PDK1/PKC signaling pathways, and iv) potential mitochondrial impairment due to the imbalance of Prohibitin (Phb) complex. Interestingly, a cross-disease study also pointed out that Phb subunits are differentially modulated in the OB across AD-related co-pathologies, providing mechanistic clues to the intriguing divergence of AD pathology across different types of dementias.

## Results

### Proteostasis impairment in the OB during AD progression

**Figure 1:**
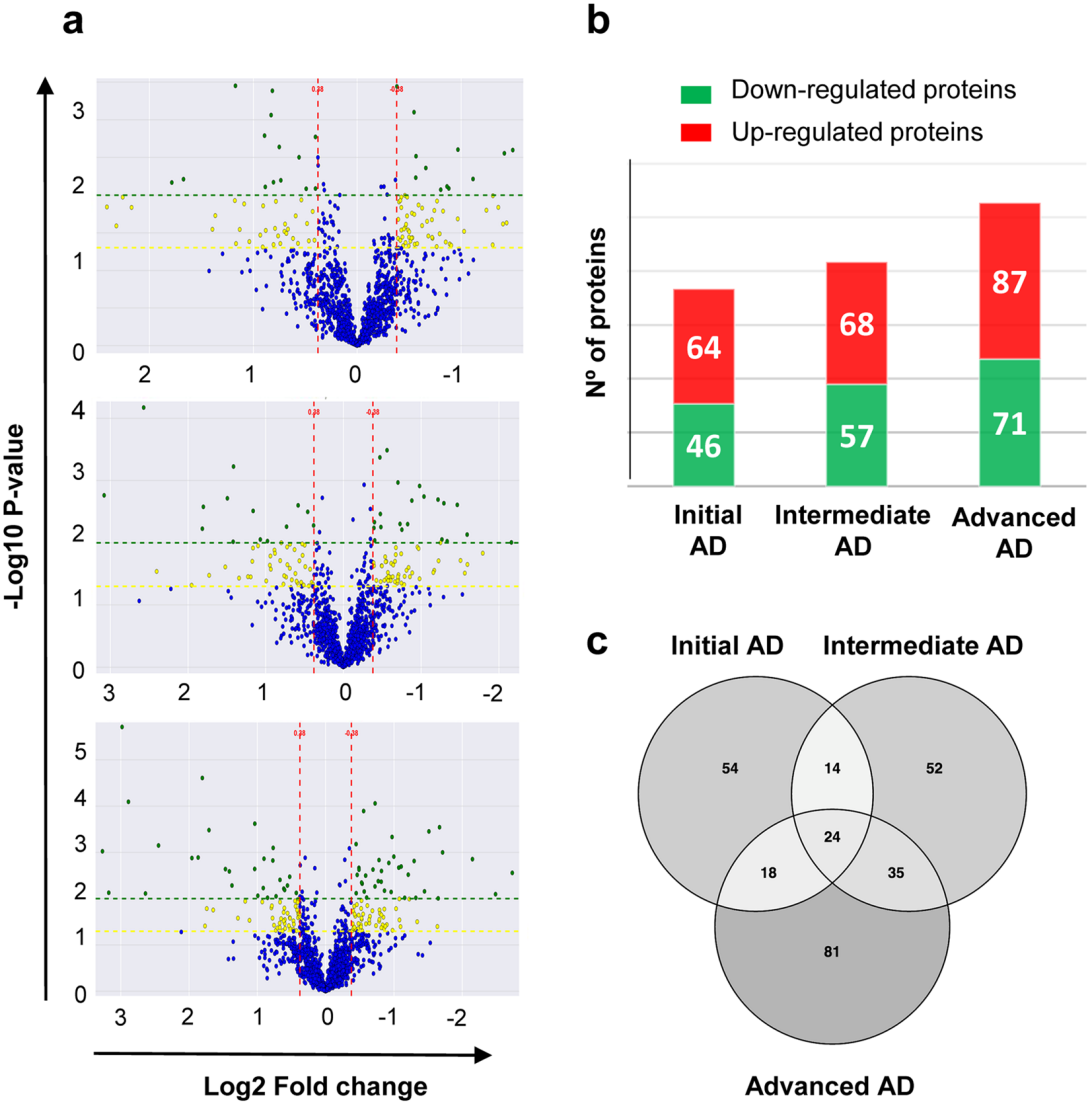
Differentially expressed proteins in the OB across AD-related phenotypes. (**a**) Volcano plots representing the fold-change of identified proteins with associated P values from the pair-wise quantitative comparisons of control vs initial AD stage (upper panel), control vs intermediate AD stage (middle panel), and control vs advanced AD stage (lower panel). In green, very significantly changed proteins (P < 0.01), in yellow, significantly changed proteins (P < 0.05) and in blue, unchanged proteins between the pair-wise comparisons. (**b**) Differential olfactory proteome distribution across AD stages. (**c**) Venn diagram of common and unique differential proteins between AD stages. The distribution of common and distinct proteins in initial, intermediate, and advanced stages is shown.

To determine the OB site-specific proteomic signature during AD progression, a label-free MS-based approach was performed on OB tissue derived from AD subjects with different grading and controls with no known neurological history (Table 1). Among 1311 quantified proteins across all experimental groups, 278 proteins tend to be differentially expressed between controls and AD phenotypes (Fig. 1a, Supplementary Table 1 and Supplementary Fig. 1). Our analysis revealed that 110 olfactory proteins are differentially expressed in early AD stages, increasing the proteome alterations as the disease progresses (125, and 158 differential proteins in intermediate and advanced stages respectively) (Fig. 1b). The distribution between up-regulated and down-regulated proteins was very similar across AD grading (Fig. 1b). Interestingly, 24 proteins overlapped between all stages (Fig. 1c), suggesting a potential role during AD evolution (Table 2). This set of proteins mainly clustered in specific biological process like growth of neurites (CLASP2, CPNE1), long-term potentiation (PPP1R1B), protein degradation (USP7, PSMD12, PSMF1), neuritogenesis (TNIK, S100B, STMN1), morphology of the nervous system (MUT, YES1), and synaptic plasticity (AP2S1, AP3D1, STXBP1). In order to evaluate the impact of AD in the OB at synaptic level, we have compared the OB differential expressed proteomes across AD staging with the information stored in three repositories containing the largest number of synapse specific proteins (G2Cdb, Synaptome DB, and SynsysNet)^16–18^. The analysis revealed that 162 out of 278 differential proteins (58% of the differential protein set) tend to localize to synaptic terminal (63, 70, and 96 differentially expressed synaptic proteins in initial, intermediate, and advanced stages respectively) (Supplementary Fig. 2). This meta-analysis verified a progressive synaptic degeneration at the level of OB during AD progression.

**Table 1:** Subjects included in the proteomic study. The neuropathological assessment was performed according to Thal phases, CERAD score, NIA-AA guidelines and PART criteria. Aβ immunopositivity was scored on a 4-tiered scale as: (−) negative, (+) 1–2 isolated Aβ depositions, (++) 3–4 Aβ depositions, and (+++) >4Aβ depositions. Graduation of phospho-TAU deposit: (−) negative +: low; ++: intermediate; +++high. PMI: post-mortem interval; n.d: not determined; MP: Mature plaques; DP: Diffuse plaques; v.d: vascular disease.

*Cases*	*age*	*sex*	*Duration*	Brain	*PMI*	*Pathological diagnosis*	*IHQ: Aβ in OB*		*IHQ: TAU in OB*	
			*(years)*	*weight (g)*	*(hours)*	*(NIA-AA) criteria*	*MP*	*DP*	*Tangles*	*neurites*
***Advanced AD***										
BCN349	70	M	4	1104	2,5	AD (A3B3C3)	++	+++	++	+++
BCN367	89	M	13	1015	3	AD (A2B3C3)	+	+++	+++	+++
BCN369	86	M	8	973	2,5	AD (A3B3C3)	+	−	+	++
BCN376	93	M	3	1050	2,4	AD (A3B3C3)	+	+++	+++	+++
***intermediate AD***										
BCN0104	85	M	12	1115	3,3	AD (A2B2C2)	−	+	+++	+++
BCN0136	97	F	9	900	n.d	AD (A2B2C2)	n.d	n.d	n.d	n.d
BCN381	77	M	17	1103	1,5	AD (A2B2C1)	−	−	++	++
BCN222	86	F	9	1000	3	AD (A2B2C2)	−	+	++	++
***Initial AD***										
BCN342	88	M	1	1400	3,45	AD (A2B1C2)	++	+	++	++
BCN336	85	F	8	1130	2	AD (A2B1C1)	−	−	+	+
BCN358	80	M	5	1090	3	AD (A2B1C1)	++	++	++	+++
A12/0046	75	F	n.d	1125	6	AD (A1B1C1)	−	−	+	+
A12/0067	72	F	n.d	810	4	AD (A1B1C1)	−	−	+	+
***Control***										
BCN362	72	M		1407	9	Thal 1 Cerad 1 no tau deposit	−	−	−	+
BCN283	99	M		992	3	No protein deposit+v.d	−	−	−	+
BCN387	81	F		1176	3,3	PART (Braak I)+v.d	−	−	−	+

**Table 2:**
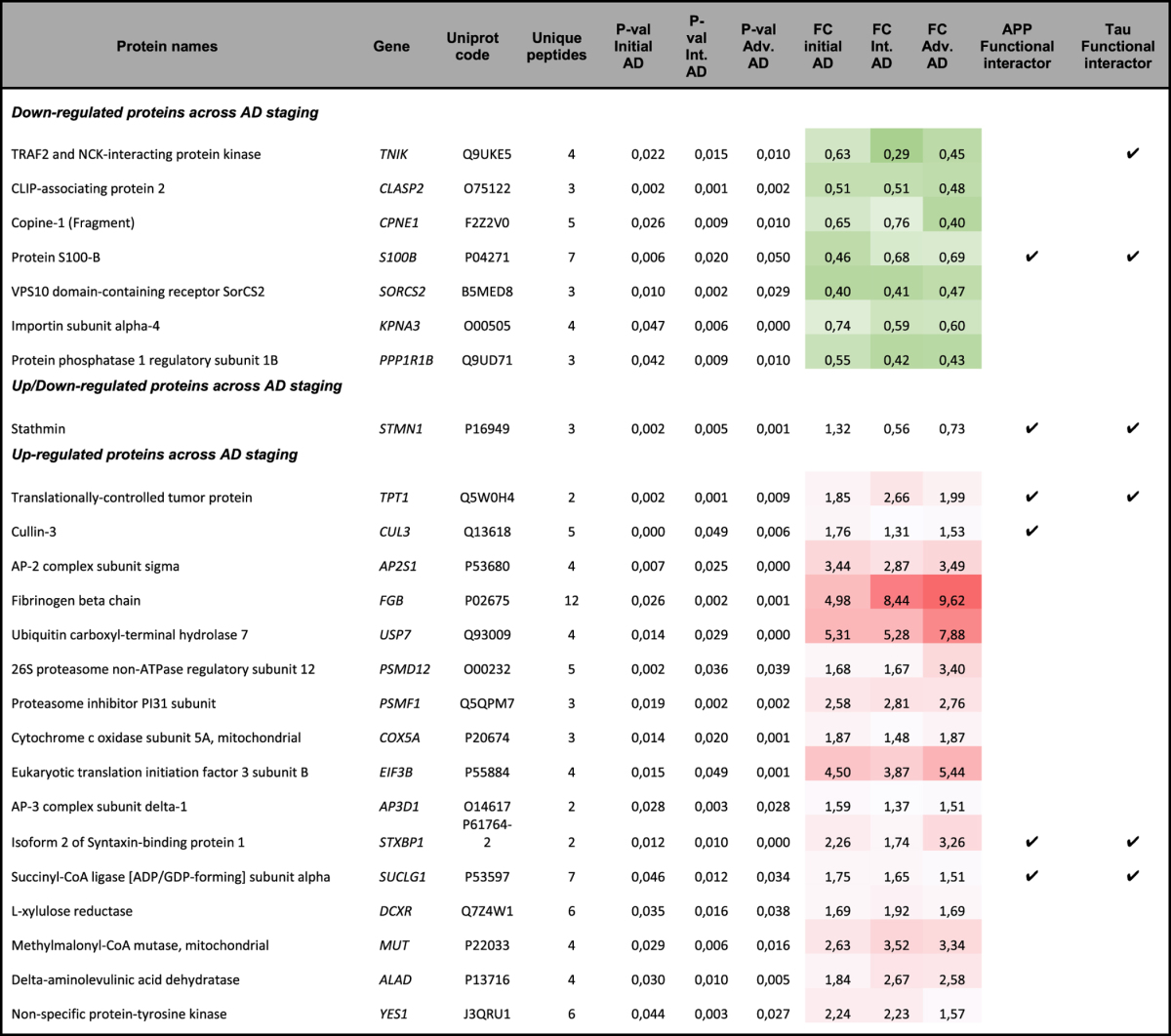
Common differential expressed OB proteins across AD stages. The fold-change (FC) of differential proteins with associated p-values from the pair-wise comparisons of control vs each AD stage, together with Protein/gene names, protein code by Uniprot, and unique peptides used for quantitation are shown. The significant downward or upward trend is represented in green or red color respectively. Proteins that are functional interactors of APP and Tau proteins are also indicated.

### Pathway-specific alterations during AD progression

**Figure 2:**
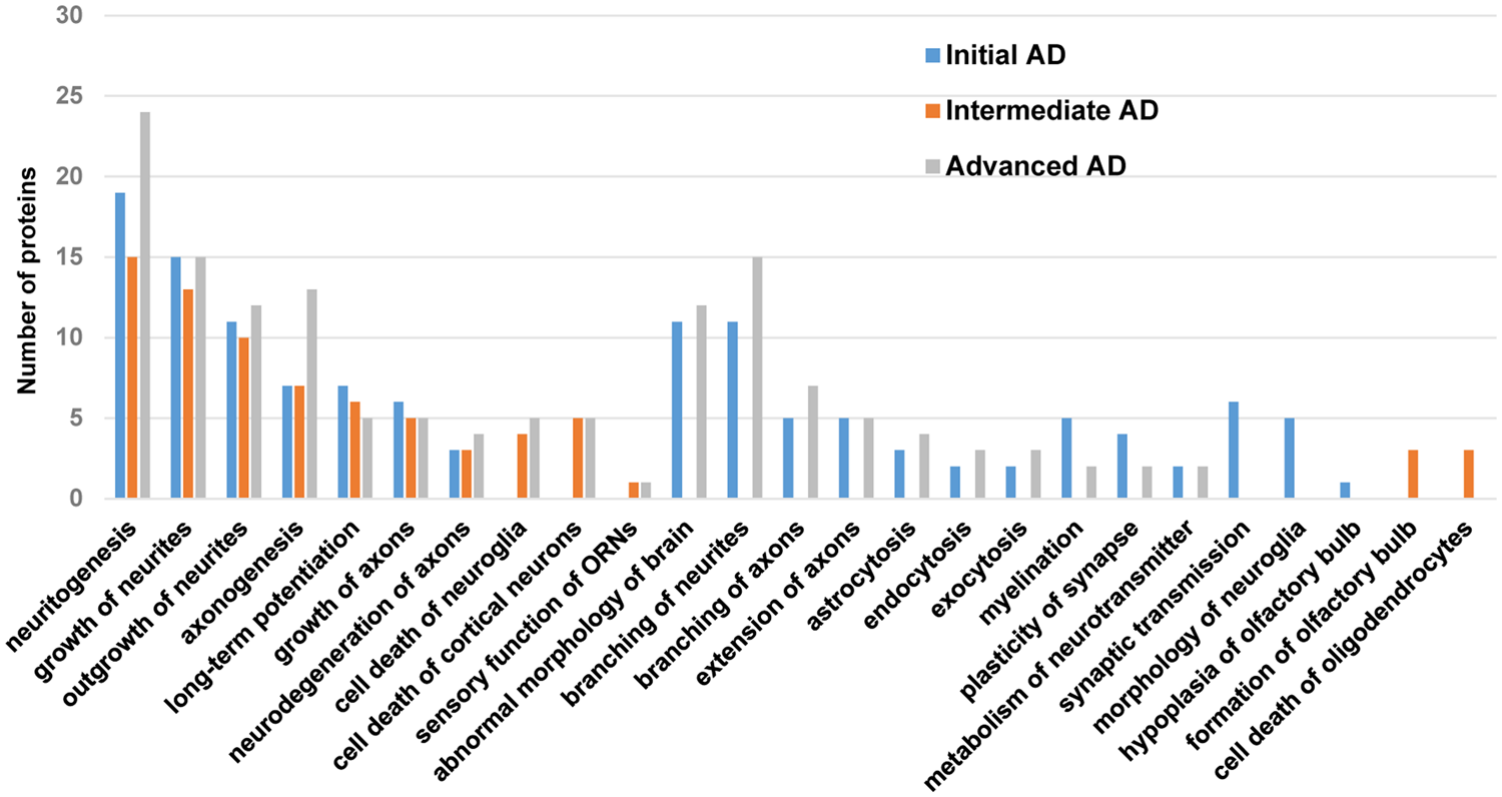
Functional metrics of the differential OB proteome across AD staging. Specific-neuronal pathway analysis for the differential OB proteomic expression profile detected in each AD stage is shown.

In order to perform a proteome mapping analysis of the stage-dependent protein profiles across specific-neuronal processes, we used the IPA information of experimental and predictive origin regarding central nervous system, in order to be confident about the potential affected signaling pathways. As shown in Fig. 2, neuronal processes such as neuritogenesis, growth and outgrowth of neurites, axonogenesis, and growth of axons are compromised across AD stages. Moreover, our results pointed out a stage-dependent deregulation of specific biological processes (Fig. 2 and Supplementary Table 2). Protein clusters involved in synaptic transmission, and morphology of neuroglia were specifically mapped in initial stages while protein groups involved in formation of OB and cell death of oligodendrocytes were exclusively detected in intermediate stages. Interestingly, a de-regulation in protein clusters related to branching of neurites and axons, astrocytosis, vesicle trafficking, and myelination appears throughout initial and advanced stages (Fig. 2).

### Network-driven proteomics reveals an imbalance in the olfactory MKK3-6/p38 MAPK and PDK1/PKC signaling across AD grading

**Figure 3:**
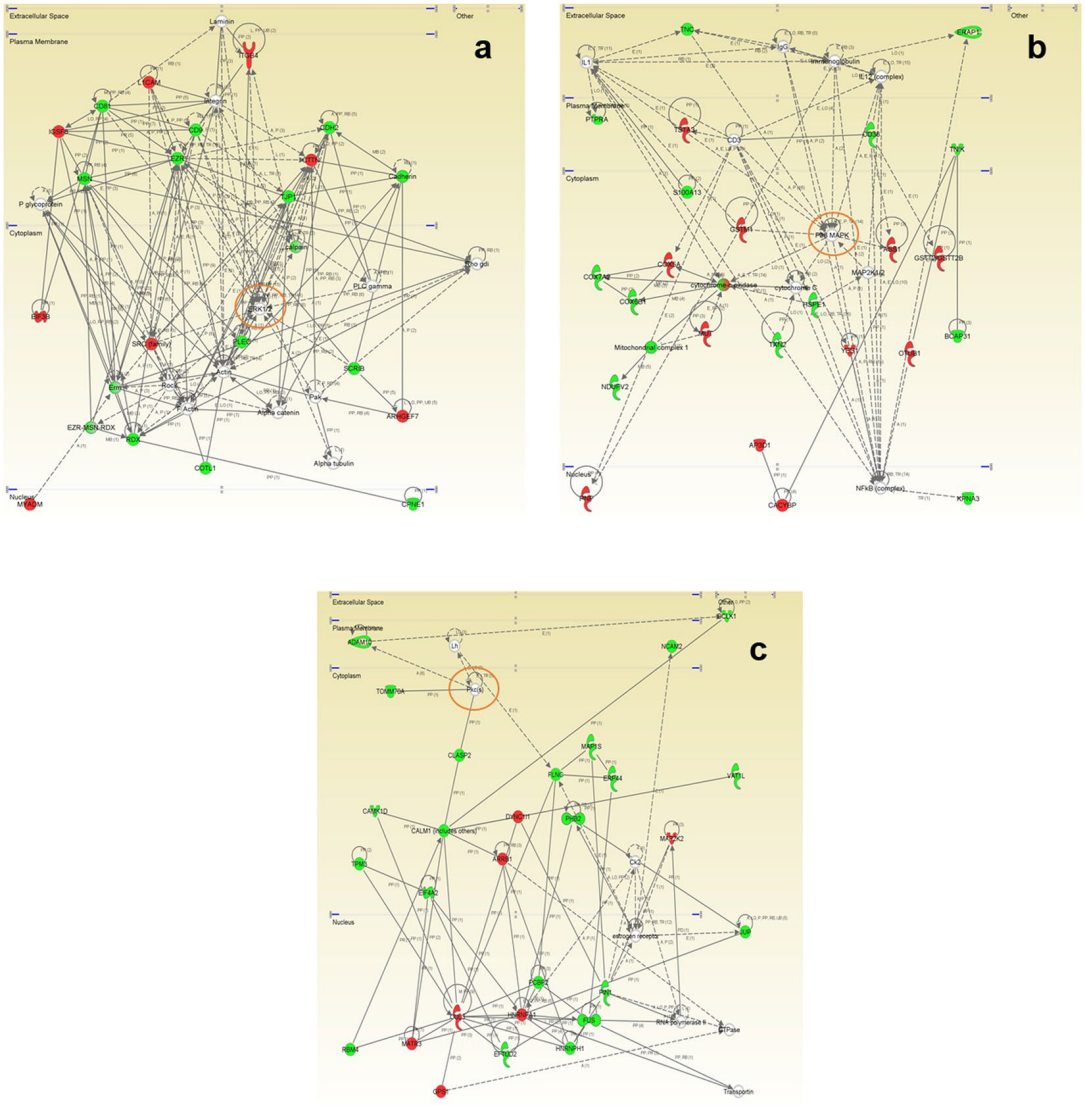
High-scoring protein interactome maps for differentially expressed proteins in the OB during AD progression. Visual representation of the relationships between differential expressed proteins and functional interactors in initial (**a**), intermediate (**b**), and advanced AD stages (**c**). Dysregulated proteins are highlighted in red (up-regulated) and green (down-regulated) for each stage. Continuous and discontinuous lines represent direct and indirect interactions respectively. The complete legend including main features, molecule shapes, and relationships is found in http://ingenuity.force.com/ipa/articles/Feature_Description/Legend.

**Figure 4:**
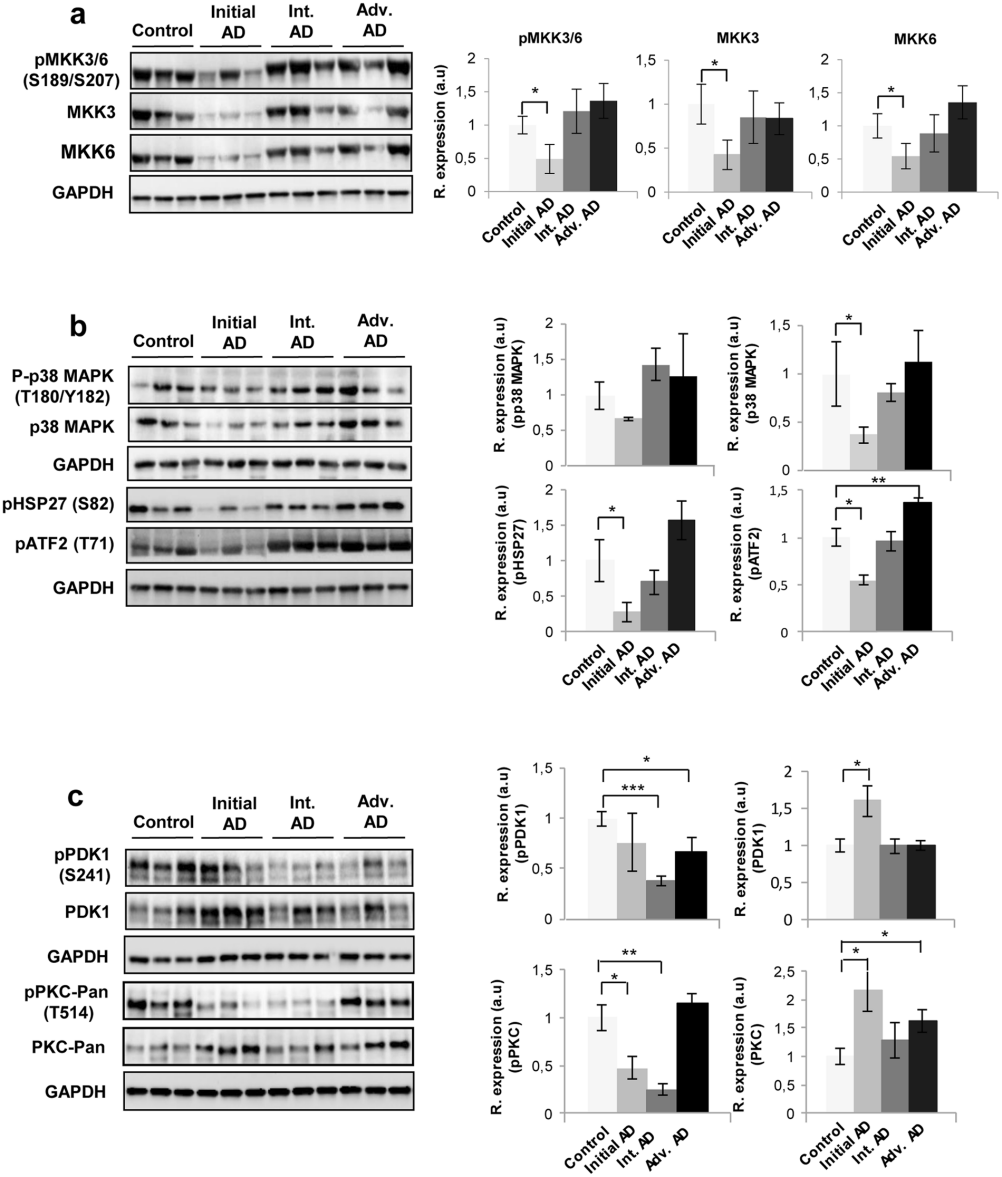
Signaling pathways disrupted in the OB across AD grading. Levels and residue-specific phosphorylation of MKK3/6 (**a**), p38 MAPK, ATF2, and HSP27 (**b**), PDK1, and PKC (**c**) in the OB across AD phenotypes. Equal loading of the gels was assessed by Ponceau staining and hybridization with a GAPDH specific antibody. Right panels show histograms of band densities. Data are presented as mean ± SEM from 5 independent OB samples per group. *P < 0.05 vs control group; **P < 0.01 vs control group. Representative Western blot gels (n = 3/experimental group) are shown. Full-length blots/gels are presented in Supplementary information.

To enhance the analytical outcome of proteomic experiments, we have performed proteome-scale interaction networks merging the olfactory proteins that tend to be de-regulated across stages of AD. To that end, a protein interactome map has been constructed for each stage using the IPA software (Fig. 3). In initial stages, a deregulation of cross-linkers between plasma membrane and actin-based cytoskeleton such as the protein complex ezrin-moesin-radixin (EZR-MSN-RDX) and regulators of the interaction between components of cell-cell junctions (L1CAM, CD9, CD81, CTTN, cadherin) suggested an imbalance in the cellular assembly and morphology at early AD stages (Fig. 3a). The functional clustering also suggested a central function of ERK1/2 mediating the structural stabilization at the level of OB (Fig. 3a). In accordance with these data, our group has previously demonstrated an early hyperactivation of ERK1/2 in the OB of AD subjects^7^. In intermediate stages, the proteome-scale interaction network reflected an impaired mitochondrial function and an imbalance in redox signaling, due to dysregulation of subunits of mitochondrial respiratory chain complexes I, and IV (COX5A, COX7A2, COX6B1, NDUFV2) and protein components involved in antioxidant defense mechanisms (HSPE1, GSTM1, TXN2) (Fig. 3b). In advanced stages, the functional interactome network indicated an alteration in HNRNP complexes (FUS, HNRNPA1, HNRNPH1) and specific RNA binding proteins (EFTUD2, and MATR3), suggesting an impairment in RNA stability, and pre-mRNA splicing processes. Moreover, the alteration of COP9 signalosome complex subunit 1 (GPS1), and cullin-3 (CUL3) pointed out an alteration in the proteasomal degradation pathway in advanced stages of the disease (Fig. 3c). Both p38 MAPK and PKC appeared as principal nodes in protein interactome maps (Fig. 3b and c). Even though changes in their expression were not detected in our proteomic experiments, the alteration of some of their targets may be compatible with a dysregulation of their functionality during AD progression at the level of OB. Subsequent experiments were performed to monitor the activation state of p38 MAPK and PKC signaling pathways across AD stages. MKK3 and MKK6 are two closely related dual-specificity protein kinases that activate p38 MAPK^19^. Western-blot analysis revealed a decrease in the activation status of upstream MKK3 and MKK6 in initial AD stages (Fig. 4a). This early down-regulation was accompanied by a fall in p38 MAPK levels and a paralleled decrease in ATF2 and HSP27 phosphorylation (Fig. 4b), well-known downstream substrates of p38 MAPK^20, 21^. However, olfactory p38 MAPK activity tends to increase during AD progression, as demonstrated by the increment in the phosphorylation status of HSP27 and ATF2 in advanced stages (Fig. 4b). PDK1 activity depends on the autophosphorylation on Ser241 and activates PKC signal transduction by phosphorylation on the activation loop^22, 23^. Although, a significant up-regulation in total PDK1 and PKC levels was evidenced in early stages (Fig. 4c), PDK1 inactivation was accompanied by a decrease in the activation status of PKC isoforms in intermediate stages, as revealed by Western-blot using a specific antibody against phosphorylated PKC isoforms at a residue homologous to activated Thr514 of human PKCγ (Fig. 4c). Accompanying the PKC inactivation, Myristoylated alanine-rich C-kinase substrate (MARCKS), a substrate of PKC^24^ was also down-regulated in intermediate stages (Supplementary Table 1). However, the accumulation of PKC isoforms maintained PKC active in advanced stages, despite the PDK1 inactivation observed in these stages (Fig. 4c). Altogether, an early disruption in upstream p38 MAPK pathway and a subsequent impairment of PDK1/PKC signaling axis occurs in the OB from AD subjects. However, the tangled regulatory mechanisms that govern the PKC signaling needs further exploration, to elucidate the specific role of each PKC isoform during the AD neurodegeneration that occurs in the OB.

### Dysregulation of AD-related protein interactomes in the OB during the neurodegenerative process

**Figure 5:**
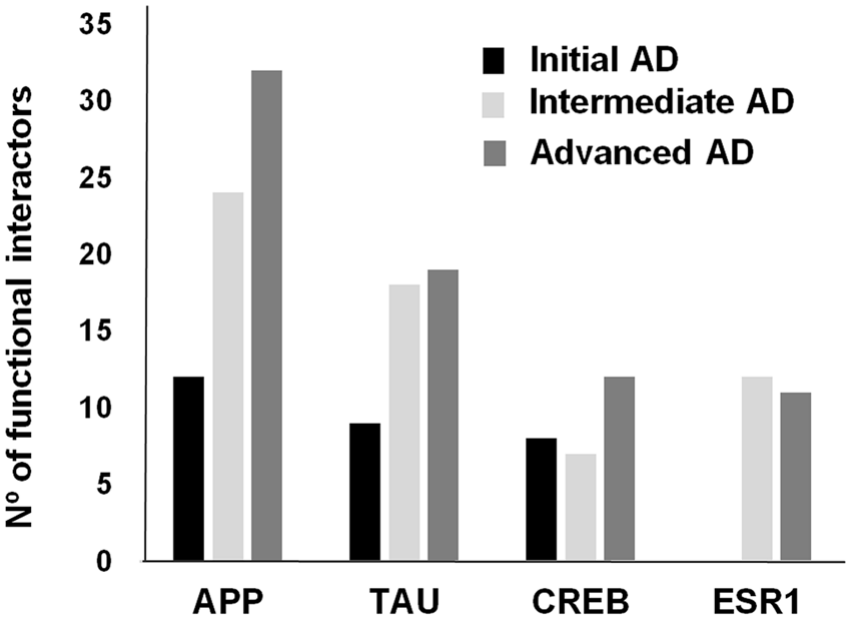
Functional interactome distribution across AD stages. The number of predicted functional interactors for hub proteins with impact in neurobiology is represented.

We consider that the discovery of unexpected relationships between apparently unrelated proteins and AD-causing neuropathological substrates is a powerful strategy for the characterization of novel AD causative/susceptibility proteins with a central role during the neurodegenerative process that occurs in olfactory areas. We explored whether well-established AD-related proteins were indeed highly interconnected with the stage-dependent differential olfactory proteomes. As shown in Fig. 5, differential functional interactors for neuropathological substrates like APP and tau proteins were identified in the OB. With respect to OB controls, the APP interactome was composed by 12, 24, and 32 differential targets in initial, intermediate, and advanced AD stages respectively (Supplementary Fig. 3). In the case of tau protein, 9 differential targets were detected in early AD stages, whereas 18 and 19 targets constituted the differential Tau interactome in intermediate and advanced AD stages respectively (Fig. 5 and Supplementary Fig. 4). Interestingly, 5 differential targets were shared between APP and Tau interactomes across AD grading. These proteins correspond to succinyl-CoA ligase [ADP/GDP-forming] subunit alpha (SUCLG1), Stathmin 1 (STMN1), translationally-controlled tumor protein (TPT1), protein S100-B (S100B), and syntaxin-binding protein 1 (STXBP1) (Supplementary Figs 3 and 4). Our analysis also revealed other functional interactomes that are modulated during AD progression in which the central nodes correspond to cAMP responsive element binding protein 1 (CREB1) and estrogen receptor 1 (ESR1) (Fig. 5 and Supplementary Figs 5 and 6).

### Validation of differential olfactory proteins across AD grading: Focus on Prohibitin complex

**Figure 6:**
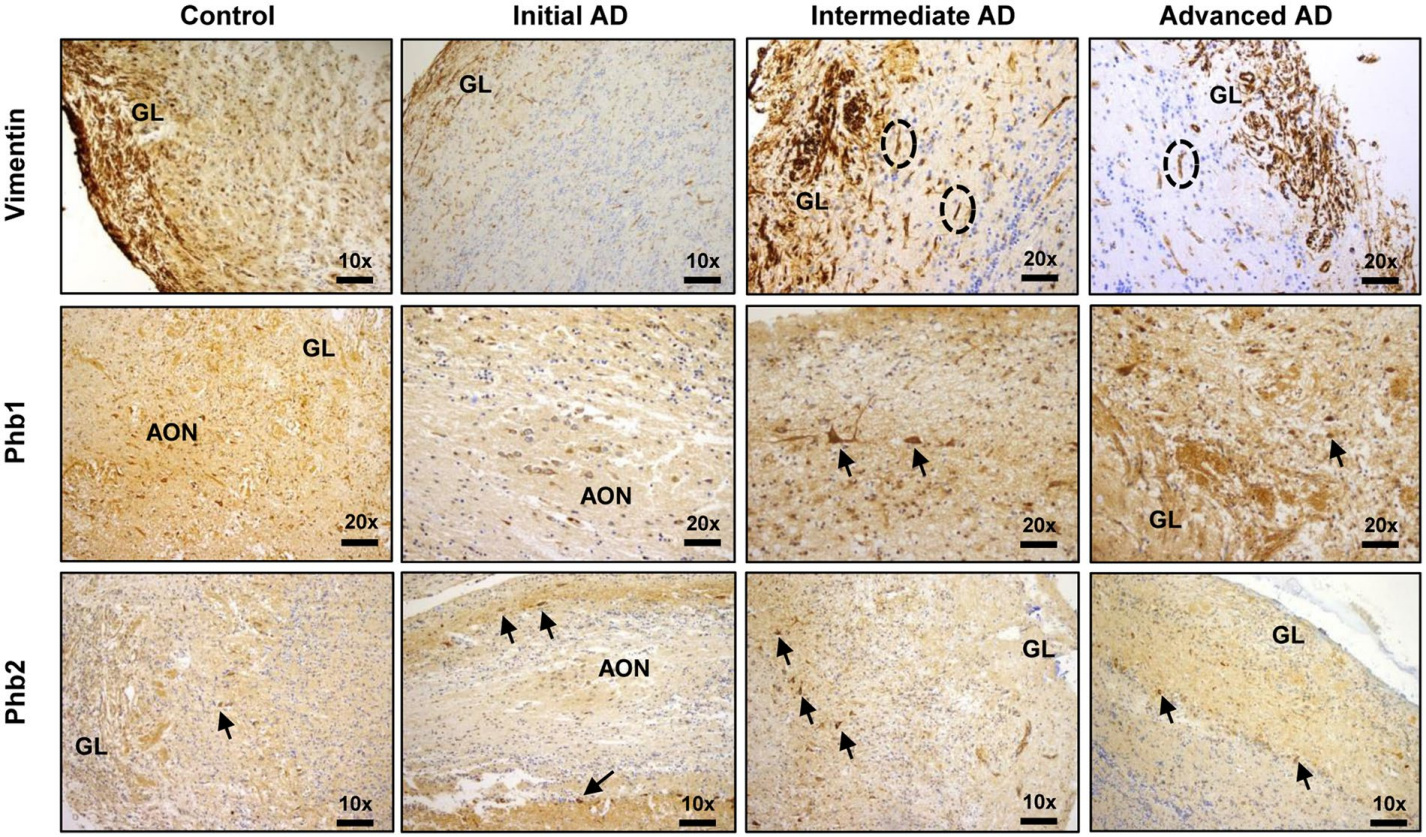
Immunohistochemical localization of OB Vim, Phb1 and Phb2 across AD grading. First line: Representative immunohistochemical staining pattern of Vim across AD grading. Positive staining in glomerular cell layer (GL) and wall of endothelial cells (ovals). Second line: Representative immunohistochemical staining pattern of Phb1 across AD grading. Positive staining in glomerular layer (GL), anterior olfactory nucleus (AON), and mitral cells (asterisks). Third line: Representative immunohistochemical staining pattern of Phb2 across AD grading. Positive staining in glomerular layer (GL), anterior olfactory nucleus (AON), and mitral cells (asterisks).

**Figure 7:**
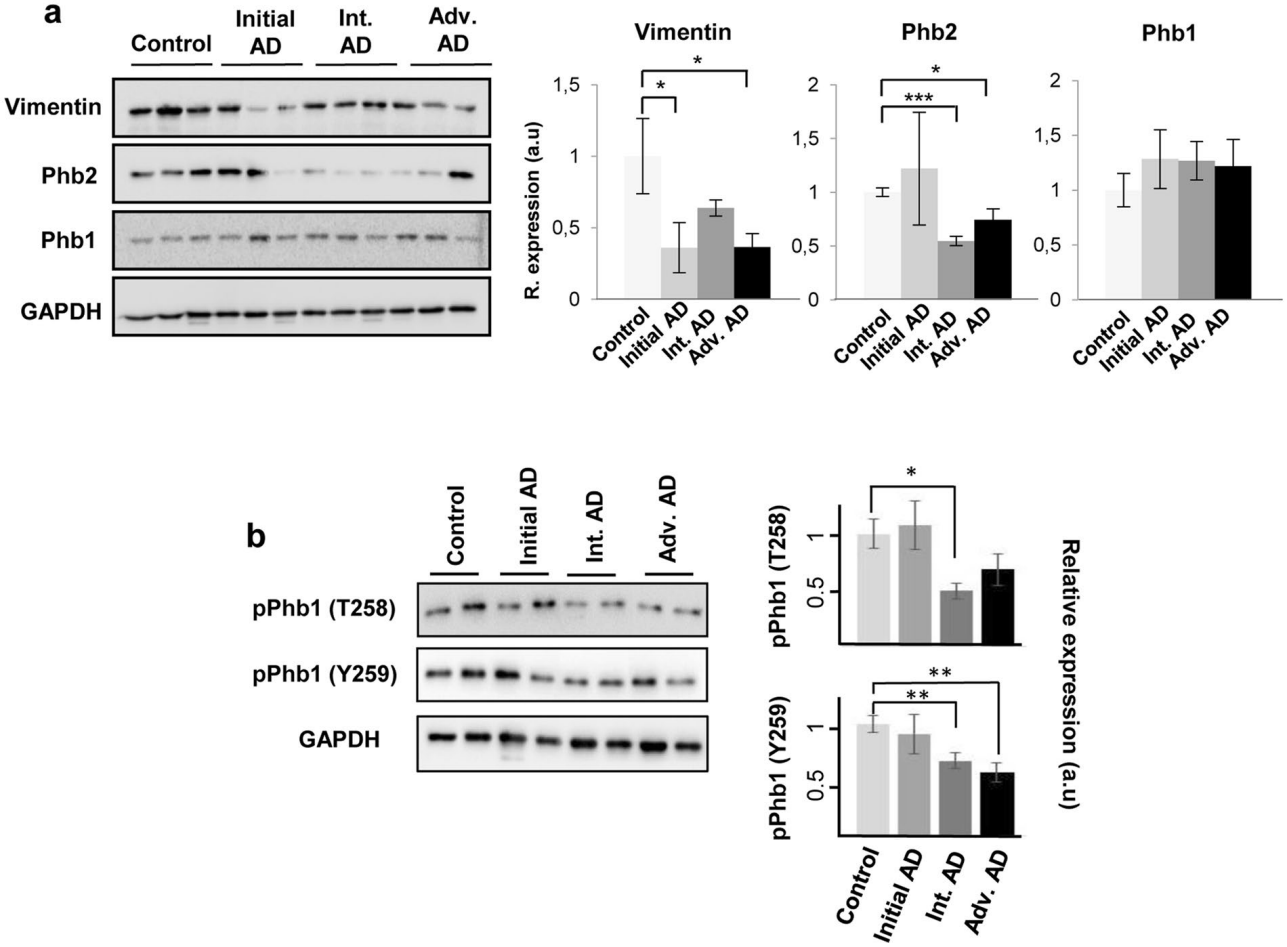
Olfactory expression of Vim and Phb subunits across AD stages. (**a**) Protein levels of Vim, Phb2, and Phb1 were monitored by Western-blotting. Equal loading of the gels was assessed by Ponceau staining and hybridization with a GAPDH specific antibody. Representative Western blot gels (n = 3/experimental group) are shown (left). Right panels show histograms of band densities. Data are presented as mean ± SEM from 3 independent OB samples per group. *P < 0.05 vs control group; ***P < 0.001 vs control group. (**b**) OB phosphorylation profiling of PHb1 across AD staging. Representative Western blot gels (n = 2/experimental group) are shown (left). Right panel shows histograms of band densities. Data are presented as mean ± SEM from 3 independent OB samples per group. *P < 0.05 vs control group; **P < 0.01 vs control group. Full-length blots/gels are presented in Supplementary information.

Previous studies identified a down-regulation of olfactory XRCC5, and FABP5 in initial AD stages, together with the overexpression of CD166 antigen, V-type proton ATPase subunit H, and histone H4 in advanced AD stages^6^. Our results confirm these previous observations (Supplementary Table 1), partially validating the label free-based liquid chromatography tandem mass spectrometry (LC-MS/MS) approach. With the aim to complement and validate quantitative proteome measurements, subsequent experiments were performed to check the steady-state levels of a subset of differential proteins using downstream assays. We consider the selection of assessing Vimentin (Vim) and Prohibitin-2 (Phb2) proteins for validation. The absence of the intermediate filament protein Vim exacerbates the amyloid plaque load and the increase in dystrophic neurites in APP/PS1 mouse model of AD^25^ and Phb2 deficiency leads to Tau hyperphosphorylation, and neurodegeneration in mice^26^. First, we performed immunohistochemical analysis to localize Vim and Phb subunits in the OB region during AD progression (Fig. 6). Vim tends to be expressed in the glomerular layer and preferentially distributed in the walls of the blood vessels (Fig. 6). Both Phb subunits were detected at dendritic connections in glomerular layer and across the neuropil, being highly expressed in the cytoplasm of mitral cells and neurons of the anterior olfactory nucleus (AON) (Fig. 6). In order to evaluate the potential role of Vim and Phb subunits in the early-affected OB region in human AD phenotypes, protein expression levels were monitored by Western blotting across AD staging (Fig. 7). In accordance with proteomic data (Supplementary Table 1), immunoblotting analysis revealed a slight decrease in olfactory Vim protein levels in initial and advanced AD stages, and a down-regulation of Phb2 protein levels in intermediate and advanced AD stages with respect to controls (Fig. 7a). Mitochondrial prohibitin complex (constituted by Phb1 and Phb2) modulates mitochondrial dynamics, participates in the mitochondrial respiratory complex assembly, and exerts beneficial effects on neurons by reducing free radical production^27, 28^. Generally, repression of Phb2 is paralleled by a concomitant reduction of its assembly partner Phb1 and vice versa^26, 29^. In agreement with our mass spectrometry (MS) data (Supplementary Table 1), Phb1 levels were unchanged across AD stages (Fig. 7a), pointing out that Phb subunits are not functionally interdependent in the OB during AD neurodegeneration. However, phosphorylated Phb1 isoforms at Thr258 and Y259 were down-expressed in intermediate and advanced AD stages (Fig. 7b), suggesting potential fluctuations in the Phb1 interactome across AD stages.

### OB protein expression of Prohibitin complex across Alzheimer-related co-pathologies

**Figure 8:**
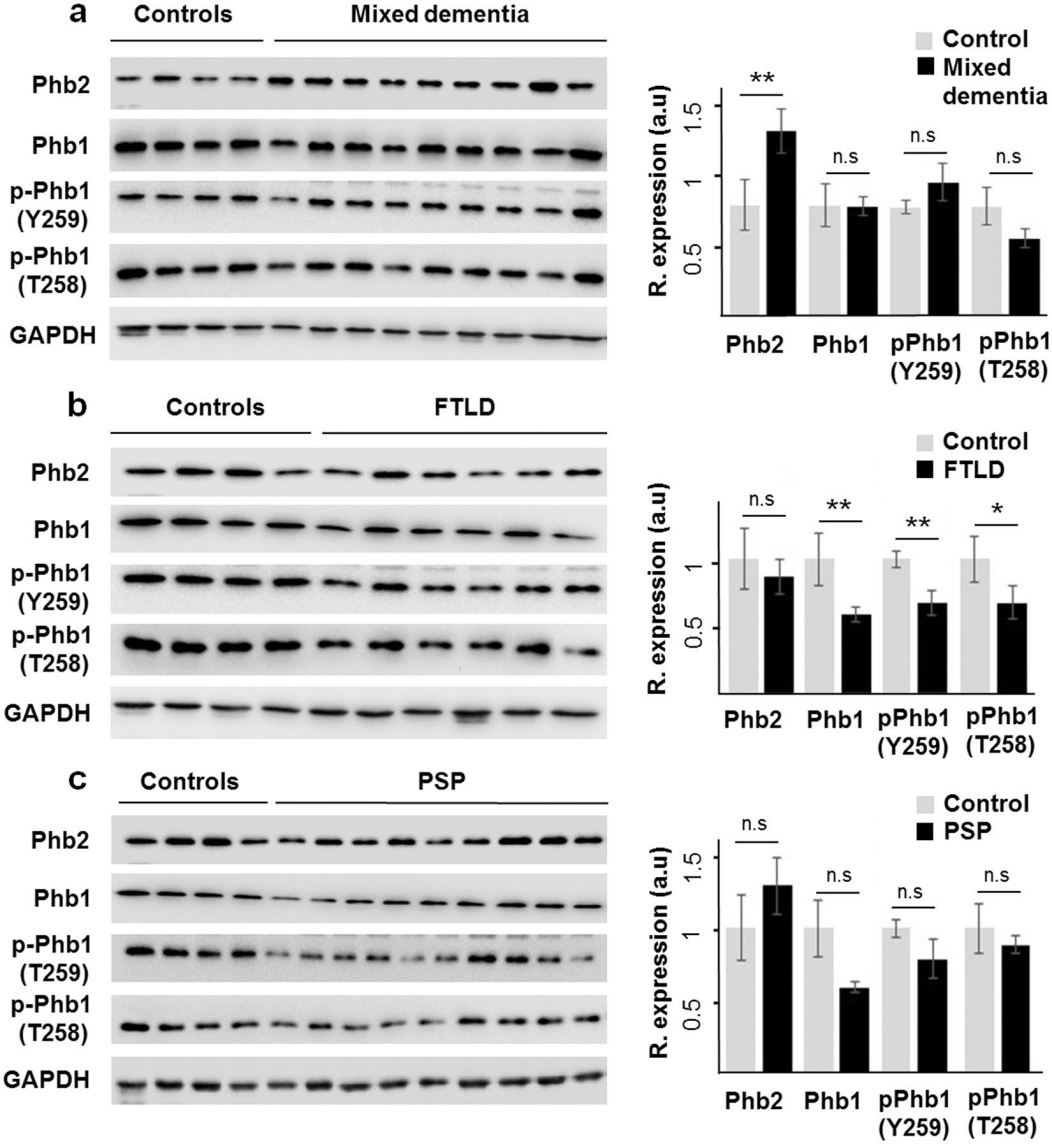
OB protein expression of Phb isoforms across AD-related proteinopathies. Olfactory expression of Phb2, Phb1, and Phb1 phosphorylated isoforms in Mix AD VD (**a**), FTLD (**b**), and PSP (**c**). Representative Western blot gels are shown for each Phb subunit. Histograms of band densities derived from 28 independent OB samples. Data are presented as mean ± SEM from: Controls (n = 4 cases), mixed dementia (mix AD VD) (n = 9 cases), FTLD (n = 6 cases), and PSP (n = 9 cases). *P < 0.05 vs control group; **P < 0.01 vs control group. Full-length blots/gels are presented in Supplementary Information.

In contrast to the common separate investigation of neurological disorders, targeted cross-disease studies comparing shared molecular relationships may give new insights into possible olfactory perturbations common for all or some neurological backgrounds. To check the potential vulnerability of PHB complex across different Alzheimer-related co-pathologies at the level of OB, we have evaluated the OB protein expression of Phb2, Phb1, Thr258-, and Y259-phosphorylated Phb1 isoforms by Western-blot across AD-related diseases (n = 28 OB samples) (Supplementary Table 3). We have included pathologies with common smell impairment like FTLD^3^, PSP where olfactory loss occurs to a lesser extent or is absent^3, 30^, and mixed dementia (Mix AD VD). Mixed dementia is a condition in which AD and vascular dementia occur at the same time, and both separate disorders often display olfactory dysfunction^31^. As shown in Fig. 8a, steady-state and phosphorylated levels of Phb1 remained unchanged in the OB from Mixed AD VD subjects respect to controls, while Phb2 protein expression is significantly increased. Moreover, a significant reduction in Phb1 expression (steady-state and phosphorylated levels) was detected in FTLD subjects, and no significant differences were observed with respect to Phb2 protein levels (Fig. 8b). On the other hand, both Phb subunits were statistically unchanged in PSP subjects, albeit 50% of PSP subjects presented a slight tendency to down-regulation in the case of Phb1 (Fig. 8c). Although equivalent olfactory deficits are observed between some AD-related co-pathologies, these data pointed out that the olfactory pattern of Phb subunits is proteinopathy-dependent, suggesting different mechanistic clues to the neurodegenerative process that occurs in the OB.

## Discussion

During the last years, proteomics has emerged as a large-scale comprehensive approach to characterize and quantify specific olfactory-related proteomes in ageing and neurodegeneration^11^. Due to the early involvement of the olfactory dysfunction in AD^4^, we consider that the application of tissue proteomics in the OB is an ideal approach that allows zooming-in where pathophysiological changes are taking place. In the current work, we have used a discovery platform combining neuropathological diagnosis, label-free quantitative proteomics, physical and functional interaction data, and biochemical approaches in order to determine the chronologic regulation of the OB proteome during AD progression. Our group has previously identified common and distinct olfactory targets across tauopathies and synucleopathies using a labeling proteomic approach^6^. The use of labeling strategies usually results in the identification of more proteins, but label-free methods allow us to analyze and compare more samples individually, indicating that both workflows are complementary^32^. It is important to note that due to technical reasons, only the most abundant OB proteins were explored. Consequently, alterations other than those reported in this study might also participate in the AD neurodegeneration at the level of the OB. However, according to our integrative meta-analysis (Supplementary Table 4), most of the differential OB proteins reported in this study has not been previously reported in differential proteomic studies performed in human AD brains.

In agreement with previous studies performed in the APP/PS1 mouse model of AD^14^, our data revealed a stage-dependent synaptic proteostasis impairment in the OB during AD pathogenesis, where more than 50% of the differential proteome across AD phenotypes tends to localize to synaptic ending. Interestingly, 17% of the OB differential proteome (47 out of 278 proteins), is also deregulated in hippocampal tissue during AD progression^12^, suggesting a coordinated regulation of specific protein modules across AD-related brain structures that might turn out detrimental or potentially protective mechanisms during the disease evolution. The synaptic plasticity imbalance was accompanied by specific proteomic fingerprints that are dynamically modulated in a stage-dependent manner throughout the OB. Interestingly, 24 early-affected proteins involved in protein degradation, growth of neurites, long-term potentiation, and neuritogenesis were deregulated across all stages, highlighting their potential importance for targeting AD at an early stage. Assuming that causative and susceptibility proteins tend to be highly interconnected in AD^33^, we have employed network-driven proteomics to yield novel insights into the signaling pathways that govern the evolution of AD at olfactory level. Our data point out that functional protein interactomes and specific pathways are dynamically modulated across AD staging in the OB, emphasizing the potential impact of stage-dependent analysis using high-throughput proteome screenings^12^. It has been proposed that the aberrant regulation of a subset of kinases may represent the triggering events leading to the spread of an aberrant signaling in AD^34^. In this context, the dysregulation of kinases regulating neuronal plasticity, learning, and memory have been proposed as the starting signal, which promotes neurotoxic outcomes^34, 35^. Analyzing the upstream signaling interactions of the differentially expressed proteomes in the OB, we determined upstream regulators (ERK1/2, p38 MAPK, and PKC) that were highly interconnected with downstream regulated proteins. Despite ERK1/2 is a well-defined pro-survival factor, neuronal ERK has been reported to be involved in the induction of cell death, APP processing, and Tau phosphorylation^36–38^. Our data point out that olfactory ERK1/2 hyperactivation^7^ may be involved in the improper cytoskeletal coupling that occurs in olfactory neurons at early stages, probably driving the synaptic impairment^39^. p38 MAPK is a multifunctional kinase in AD pathophysiology^40^. It has been shown that p38 MAPK is activated by Aβ in cultured neurons^41^, mediates the inflammatory activation initiated by Aβ^42^, and phosphorylates Tau protein^43, 44^. In this regard, previous studies have demonstrated that p38 MAPK signaling cascade is overactivated in hippocampal and cortical regions at early stages of AD pathology^45–47^, being considered as a potential target to treat AD neurodegeneration^40^. In contrast, we detected a specific upstream and downstream disruption in p38 MAPK pathway in early stages at the level of the OB, recovering normal levels in intermediate and advanced stages. This early deregulation was further validated by paralleled decrease in ATF2 and HSP27 phosphorylation, well-known downstream substrates of p38 MAPK^20, 21^. In spite of detrimental effects of p38 MAPK activation during AD pathogenesis^40^, recent studies have dissected the role of specific p38 MAPK isoforms identifying different functions. It has been characterized that the inhibition of neuronal p38alpha, but not p38beta MAPK, provides neuroprotection in different neurotoxic environments^48^. Interestingly, a depletion of neuronal p38alpha MAPK attenuates Aβ pathology in AD mouse and cell models^49^. In line with these findings, the early down-regulation of p38 MAPK in olfactory neurons might be part of the neuroprotective mechanisms induced in initial stages against Aβ. In addition, a subsequent impairment of PDK1/PKC signaling axis occurs in the OB from AD subjects. An increment in PDK1 activity has been reported in the brain from AD patients^50^, however, its inhibition or silencing points out a beneficial effect in AD-like pathology. *In vitro* studies have demonstrated that inhibition of PDK1 blocks neuronal cell death induced by Aβ^51^. Interestingly, quenching PDK1 activity in three APP-transgenic mouse models of AD rescued TACE-mediated neuroprotective cleavage of APP, and decreased Aβ deposition, counteracting memory and cognitive deficits^50^. Thus, our data suggest that the significant reduction in PDK1 activation may be a signal to protect neuronal function in the OB. PDK1 is an upstream regulator of some PKC family members^52^, critically involved in memory acquisition and maintenance^53^. Deficits in neuronal PKC signal cascades are one of the earliest abnormalities in AD brains^54^. Aβ can directly inhibit PKC isoforms, induces PKC degradation, and reduces PKC-mediated phosphorylation and membrane translocation^55–57^. In line with these findings, the PKC inactivation observed in the OB, could contribute to the Aβ-related toxicity across initial-intermediate phases of AD. Although PKC activation has been proposed for the treatment of dementias^58^, the tangled regulatory mechanisms that govern the PKC signaling needs further exploration at olfactory level, in order to clarify the specific role of each PKC isoform during the neurodegenerative process^53^.

Our proteomic screening further suggested an impairment of mitochondrial function in the OB^6^, revealing dysregulation of 29 mitochondrial proteins across intermediate and advanced AD stages. This impairment is a common finding in human AD brains, and also in rodent, and cellular AD models^59^, where intracellular Aβ accumulation leads to a decrease electron transfer efficiency, reduce ATP production, and increase ROS production^60^. Interestingly, cross-validation by immunoblotting analysis revealed a down-regulation of Phb2 protein levels in intermediate and advanced stages. Phb complex (constituted by Phb1 and Phb2) is located in the mitochondrial inner membrane acting as a membrane-bound chaperone involved in the correct folding and assembly of some of the components of the mitochondrial respiratory chain^61^. According to this hypothesis, a deficiency in Phb2 may impair the native and functional organization of respiratory proteins, compromising mitochondrial functionality^28^. Interestingly, neuron-specific deletion of Phb2 induces an aberrant mitochondrial ultrastructure, and Tau hyperphosphorylation in mice, leading to behavioral impairments and cognitive deficiencies^26^. Therefore, Phb2 deficiency might suggest a direct link between mitochondrial defects and tau pathology in olfactory neurons. We have observed a reversed Phb2 pattern between mixed dementia (Mix AD VD) respect to the protein profile observed in AD. Transcriptional and translational events may explain the difference observed in Phb2 protein levels. A possible explanation is that the vascular damage may induce a decrease in the Phb2 degradation rate at mRNA and/or protein levels at the level of the OB in mix AD VD. However, we should consider that the activation/inhibition of the transcription factor machinery that regulates the transcription of *PHB2* gene may also be compromised (as a consequence of the vascular damage), leading to an increase in Phb2 mRNA and protein levels. However, additional validation studies should be conducted employing large cohorts to verify the protein expression changes observed in our sample set. Repression of Phb2 is usually paralleled by a concomitant reduction of its assembly partner Phb1 and vice versa^26^. However, in agreement with previous studies performed in frontal cortex derived from AD subjects^62^, steady-state levels of OB Phb1 was unchanged across AD grading, demonstrating that Phb subunits are not functionally interdependent in the OB during AD neurodegeneration. In addition, Phb1 and Phb2 are present in different subcellular localizations, presenting clear and distinctive functions^63^. While some associations at the plasma membrane and in the mitochondria require both Phb1 and Phb2, both proteins function independently in the nucleus, influencing the activity of multiple transcription factors^64^. However, based on our immunohistochemical analysis, most of Phb2 staining is not detected in the nucleus of olfactory neurons, suggesting that Phb2 may not be directly involved in transcriptional events. On the other hand, Phb subunits are post-translationally modified by O-linked N-acetylglucosamine, palmitoylation, transamidation, nytrosylation, and phosphorylation^65^. A decreased in phosphorylated Phb1 isoforms at T258 and Y259 was detected in the OB from AD subjects. Both residues are present in the C-terminal coiled-coil domain that is involved in protein-protein interactions, including the interaction between Phb1 and Phb2 as well as transcriptional regulation^66^. Although the kinase that phosphorylates the Y259 remains unknown, it is well documented that Akt phosphorylates Phb1 at T258 in non-neuronal contexts. Specifically, Akt may phosphorylate this residue in the cytoplasm, promoting Phb1 mitochondrial translocation^67^ or in the lipid raft domain of the plasma membrane to activate the Ras-MAPK and PI3K/Akt pathways^68^. However, previous work in our laboratory demonstrated that the activation state of Akt remains unchanged, and ERK is hyper-activated in the OB from AD subjects^7^, suggesting that OB Phb1 dephosphorylation may be due to a phosphatase action or the inactivation of a specific kinase different than Akt. Although our results serve as a foundation for new areas of investigation into the role of olfactory signaling in human AD-related co-pathologies, further work is necessary to clarify the regulatory mechanisms involved in post-translational modifications of Phb subunits, in order to understand the final effect of olfactory Phb complex on cell survival and apoptosis across proteinopathies.

## Methods

### Materials

The following reagents and materials were used: anti-GAPDH (Calbiochem), anti-MKK3, anti-MKK6, anti-phospho MKK3 (Ser189)/MKK6 (Ser207), anti-p38 MAP kinase, anti-phospho p38 MAP kinase (Thr180/Tyr 182), anti-phospho HSP27 (Ser82), anti-phospho ATF2 (Thr71), anti-PDK1, anti-phospho PDK1 (S241), anti-PKC-Pan, anti-phospho PKC-pan (T514), anti-Prohibitin 1 (Phb1), anti-Prohibitin 2 (Phb2), (Cell signaling). Anti-vimentin antibody was purchased from Santa Cruz biotechnology. Anti-phospho Phb1 (T258), and anti-phospho Phb1 (Y259) were purchased from Signalway Antibody. Electrophoresis reagents were purchased from Biorad and trypsin from Promega.

### Human samples

According to the Spanish Law 14/2007 of Biomedical Research, inform written consent forms of the Neurological Tissue Bank of Navarra Health Service, Brain Bank of IDIBELL, and Neurological Tissue Bank of IDIBAPS-Hospital Clinic (Barcelona, Spain) was obtained for research purposes from relatives of patients included in this study. The study was conducted in accordance with the Declaration of Helsinki and all assessments, post-mortem evaluations, and procedures were previously approved by the Clinical Ethics Committee of Navarra Health Service. For the proteomic phase, fourteen AD cases were distributed into different groups according to specific consensus diagnostic criteria^69–71^: initial, intermediate, and advanced AD stages (n = 4–5/group). Three cases from elderly subjects with no history or histological findings of any neurological disease were used as a control group. All human brains considered in the proteomic study had a post-mortem interval (PMI) lower than 10 hours (Table 1). Brain processing and the neuropathological study for protein deposits aggregates Aβ and phospho-Tau were performed as previously described^6^. For the discovery phase, neuropathological assessment was performed according to standardized neuropathological scoring/grading systems, including Thal phases of Beta-amyloid deposition, Braak staging of neurofibrillary lesions, Consortium to Established a Registry for Alzheimer’s Disease, National Institute on Aging-Alzheimer’s Association (NIA-AA) guidelines, and primary age-related tauopathy (PART) criteria^69–73^. For the validation phase, OB tissue from additional controls and AD subjects were included (n = 4–5/group) (Supplementary Table 3). This material was obtained from the Neurological Tissue Banks of IDIBELL and IDIBAPS-Hospital Clinic, Barcelona, Spain. For the specificity analysis, different NDs were considered: Progressive supranuclear palsy (PSP) (n = 9 cases; 4F/5M; median age: 74 years), frontotemporal lobar degeneration (FTLD) (n = 6; 3F/3M; median age: 81 years), mixed dementia (mix AD VD) (n = 9 cases; 4F/5M; median age: 85 years), and controls (n = 4; 1F/3M; median age: 79 years). In these cases, neuropathological assessment was performed according to standardized neuropathological guidelines: Mackenzie criteria for FTLD pathology^74^, NINDS-AIREN criteria for vascular dementia^75^, and NINDS criteria for PSP^76^. 80% of the OB samples included in this phase had a PMI lower than 10 hours (Supplementary Table 3).

### Sample preparation for proteomic analysis

OB specimens derived from control and AD cases were homogenized in lysis buffer containing 7 M urea, 2 M thiourea, 4% (w/v) CHAPS, 50 mM DTT. The homogenates were spinned down at 100.000 × g for 1 h at 15 °C. Prior to proteomic analysis, protein extracts were precipitated with methanol/choloroform, and pellets dissolved in 6M Urea, Tris 100mM pH 7.8. Protein quantitation was performed with the Bradford assay kit (Bio-Rad).

### Label free LC-MS/MS

The protein extract for each sample was diluted in Laemmli sample buffer and loaded into a 0.75 mm thick polyacrylamide gel with a 4% stacking gel casted over a 12.5% resolving gel. The run was stopped as soon as the front entered 3 mm into the resolving gel so that the whole proteome became concentrated in the stacking/resolving gel interface. Bands were stained with Coomassie Brilliant Blue and excised from the gel. Protein enzymatic cleavage (10 ug) was carried out with trypsin (Promega; 1:20, w/w) at 37 °C for 16 h as previously described^77^. Purification and concentration of peptides was performed using C18 Zip Tip Solid Phase Extraction (Millipore). Peptides mixtures were separated by reverse phase chromatography using an Eksigent nanoLC ultra 2D pump fitted with a 75 μm ID column (Eksigent 0.075 × 250). Samples were first loaded for desalting and concentration into a 0.5 cm length 100 μm ID precolumn packed with the same chemistry as the separating column. Mobile phases were 100% water 0.1% formic acid (FA) (buffer A) and 100% Acetonitrile 0.1% FA (buffer B). Column gradient was developed in a 240 min two step gradient from 5% B to 25% B in 210 min and 25%B to 40% B in 30 min. Column was equilibrated in 95% B for 9 min and 5% B for 14 min. During all process, precolumn was in line with column and flow maintained all along the gradient at 300 nl/min. Eluting peptides from the column were analyzed using an Sciex 5600 Triple-TOF system. Information data acquisition was acquired upon a survey scan performed in a mass range from 350 m/z up to 1250 m/z in a scan time of 250 ms. Top 35 peaks were selected for fragmentation. Minimum accumulation time for MS/MS was set to 100 ms giving a total cycle time of 3.8 s. Product ions were scanned in a mass range from 230 m/z up to 1500 m/z and excluded for further fragmentation during 15 s.

### Peptide Identification and Quantification

MS/MS data acquisition was performed using Analyst 1.7.1 (AB Sciex) and spectra files were processed through Protein Pilot Software (v.5.0-ABSciex) using Paragon™ algorithm (v.4.0.0.0) for database search^78^, Progroup™ for data grouping, and searched against the concatenated target-decoy UniProt proteome reference Human database (Proteome ID: UP000005640, 70902 proteins, December 2015). False discovery rate was performed using a non lineal fitting method^79^ and displayed results were those reporting a 1% Global false discovery rate or better. The peptide quantification was performed using the Progenesis LC−MS software (ver. 2.0.5556.29015, Nonlinear Dynamics). Using the accurate mass measurements from full survey scans in the TOF detector and the observed retention times, runs were aligned to compensate for between-run variations in our nanoLC separation system. To this end, all runs were aligned to a reference run automatically chosen by the software, and a master list of features considering m/z values and retention times was generated. The quality of these alignments was manually supervised with the help of quality scores provided by the software. The peptide identifications were exported from Protein Pilot software and imported in Progenesis LC− MS software where they were matched to the respective features. Output data files were managed using R scripts for subsequent statistical analyses and representation. Proteins identified by site (identification based only on a modification), reverse proteins (identified by decoy database) and potential contaminants were filtered out. Proteins quantified with at least two unique peptides, an ANOVA p-value lower than 0.05, and an absolute fold change of <0.77 (down-regulation) or >1.3 (up-regulation) in linear scale were considered to be significantly differentially expressed. MS raw data and search results files have been deposited to the ProteomeXchange Consortium (http://proteomecentral.proteomexchange.org) via the PRIDE partner repository^80^ with the dataset identifiers PXD005319.

### Bioinformatics

The proteomic data were analyzed through the use of QIAGEN’s Ingenuity® Pathway Analysis (IPA) (QIAGEN Redwood City, www.qiagen.com/ingenuity), in order to detect and infer differentially activated/deactivated pathways as a result of AD phenotypes. This software comprises curated information from databases of experimental and predictive origin, enabling discovery of highly represented functions, pathways, and interactome networks.

### Immunohistochemistry

For the immunohistochemical study, formalin fixed sections (3–5 mm-thick) were mounted on slides and deparaffinized. Tissue sections were labelled with the following primary antibodies: anti-vimentin (dilution 1/200), anti-Prohibitin-1 (Phb1) (dilution 1/120), and anti-Prohibitin-2 (Phb2) (dilution 1/50). The reaction product was visualized using an automated slide immunostainer (Leica Bond Max) with Bond Polymer Refine Detection (Leica Biosystems Newcastle Ltd).

### Immunoblotting analysis

Equal amounts of protein (10 μg) were resolved in 12.5% SDS-PAGE gels. OB proteins derived from human samples were electrophoretically transferred onto nitrocellulose membranes using a Trans-blot Turbo transfer system (up to 25V, 7min) (Bio-rad). Equal loading of the gels was assessed by Ponceau staining. Membranes were probed with primary antibodies at 1:1000 dilution in 5% nonfat milk or BSA. After incubation with the appropriate horseradish peroxidase-conjugated secondary antibody (1:5000), the immunoreactivity was visualized by enhanced chemiluminiscence (Perkin Elmer) and detected by a Chemidoc MP Imaging System (Bio-Rad). After densitometric analyses (Image Lab Software Version 5.2; Bio-Rad), optical density values were expressed as arbitrary units and normalized to GAPDH.

## Electronic supplementary material

Supplementary information

Related Manuscript File

Related Manuscript File

Related Manuscript File

Related Manuscript File
