# Supplementary material for: Olfactory bulb neuroproteomics reveals a chronological perturbation of survival routes and a disruption of prohibitin complex during Alzheimer’s disease progression
Source: Sci Rep. 2017 Aug 22;7:9115. doi: 10.1038/s41598-017-09481-x (PMC5567385; doi:10.1038/s41598-017-09481-x)
Supplement: Supplementary file 1 — Supplementary information [file 41598_2017_9481_MOESM1_ESM.pdf]

Olfactory bulb neuroproteomics reveals a chronological perturbation of survival routes and a dysruption of prohibitin complex during Alzheimer's disease progression

Mercedes Lachen-Montes, Andrea González-Morales, María Victoria Zelaya, Estela Pérez-Valderrama, Karina Ausin, Isidro Ferrer, Joaquín Fernández-Irigoyen, Enrique Santamaría

# - Supplementary Figures & QCs -

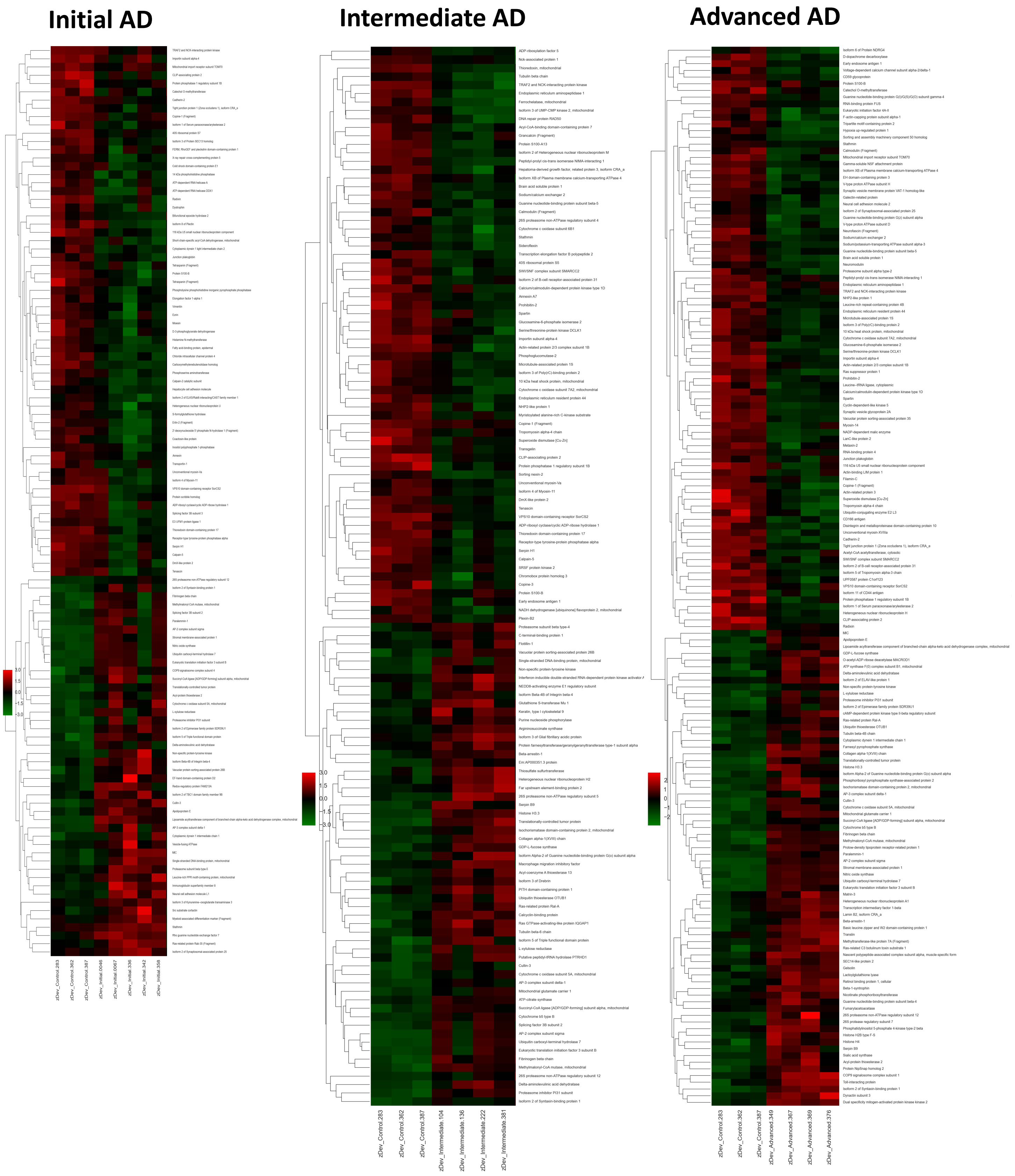

Supplementary figure 1: Heat maps representing the degree of change for the differentially expressed OB proteins ($P < 0.05$) across AD staging. Lateral Legend indicates color-coded fold-change on Log10 scale. Red and green, up- and down-regulated proteins, respectively.

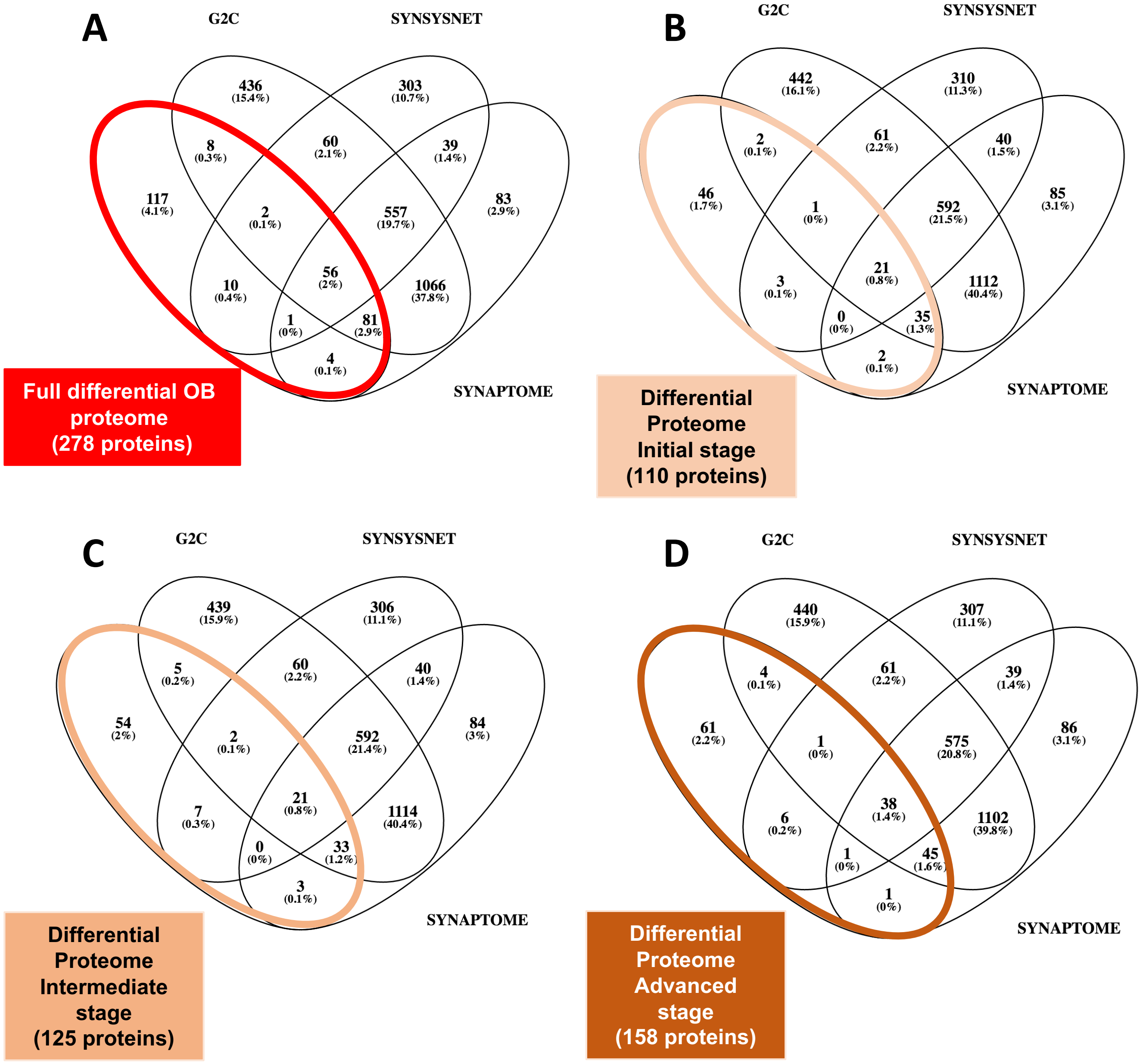

Supplementary figure 2: Intersection of proteins derived from differential proteomic expression profiling of OB in AD stages and the "synaptic proteome" composed by the three major databases on synaptic proteins (C2G, SynsysNet, and Synaptome DB). Numbers represent the number of shared proteins in the respective overlapping areas. Venn diagrams representing the overlap between the "synaptic proteome" and the full OB differential expressed proteome (A), OB differential proteome detected in initial stages (B), in intermediate stages (C), and in advanced stages (D) are shown.

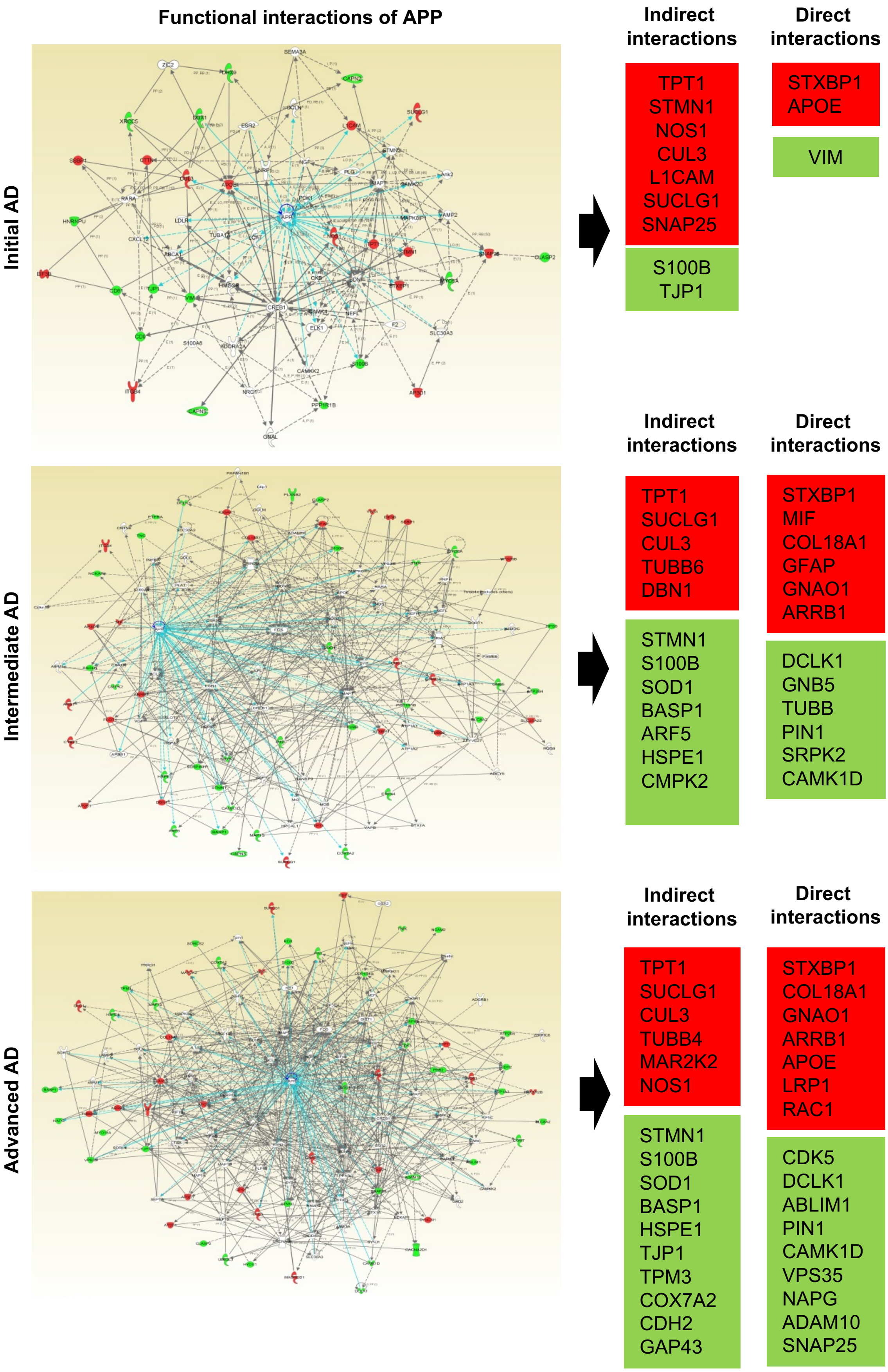

Supplementary figure 3. Modulation of APP protein interaction network during the neurodegenerative process in the OB. For each AD stage, the APP interactome is shown, where protein connections are indicated with blue lines (left panels), highlighting indirect and direct targets (right).

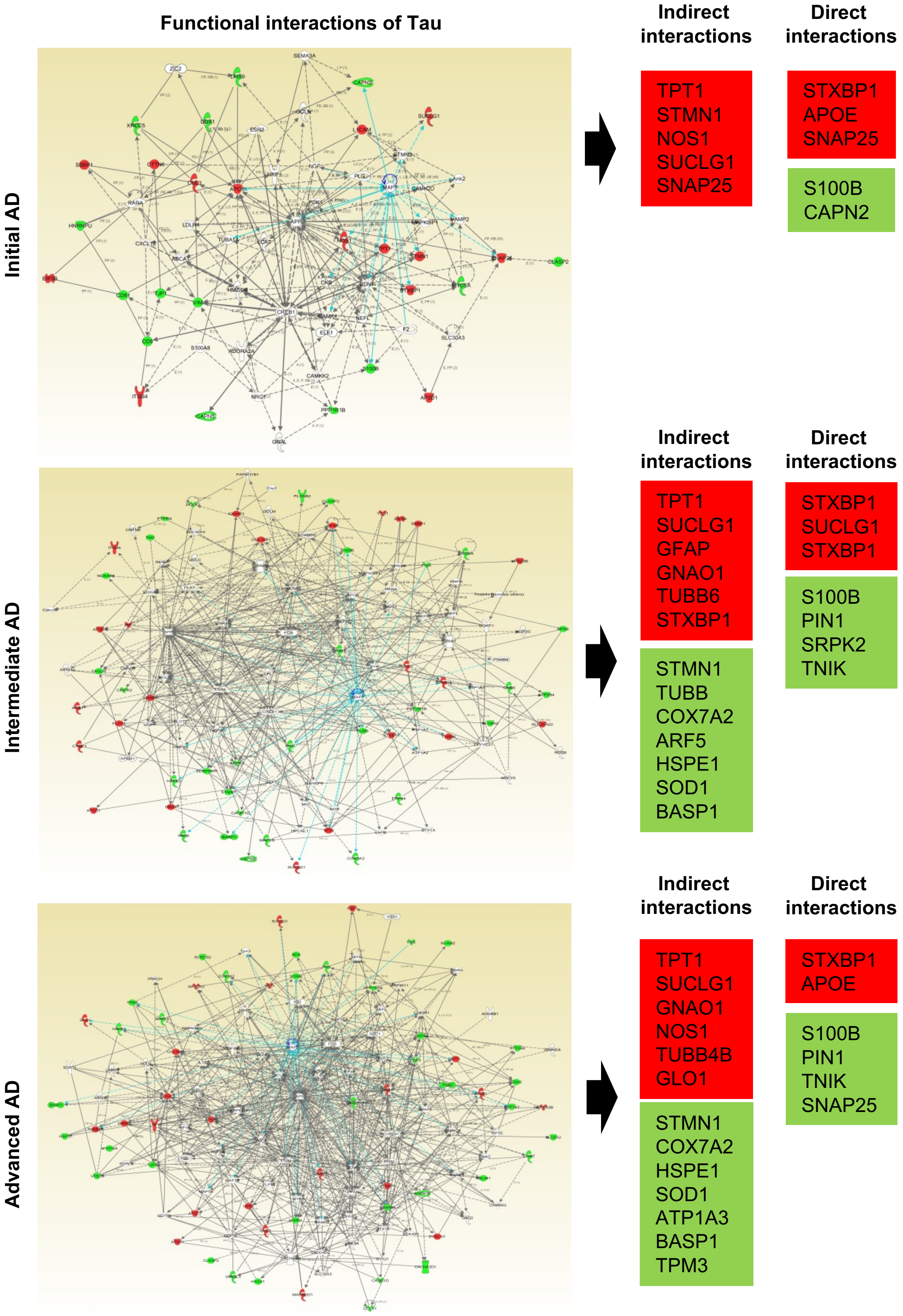

Supplementary figure 4. Modulation of Tau protein interaction network during the neurodegenerative process in the OB. For each AD stage, the tau interactome is shown, where protein connections are indicated with blue lines (left panels), highlighting indirect and direct targets (right).

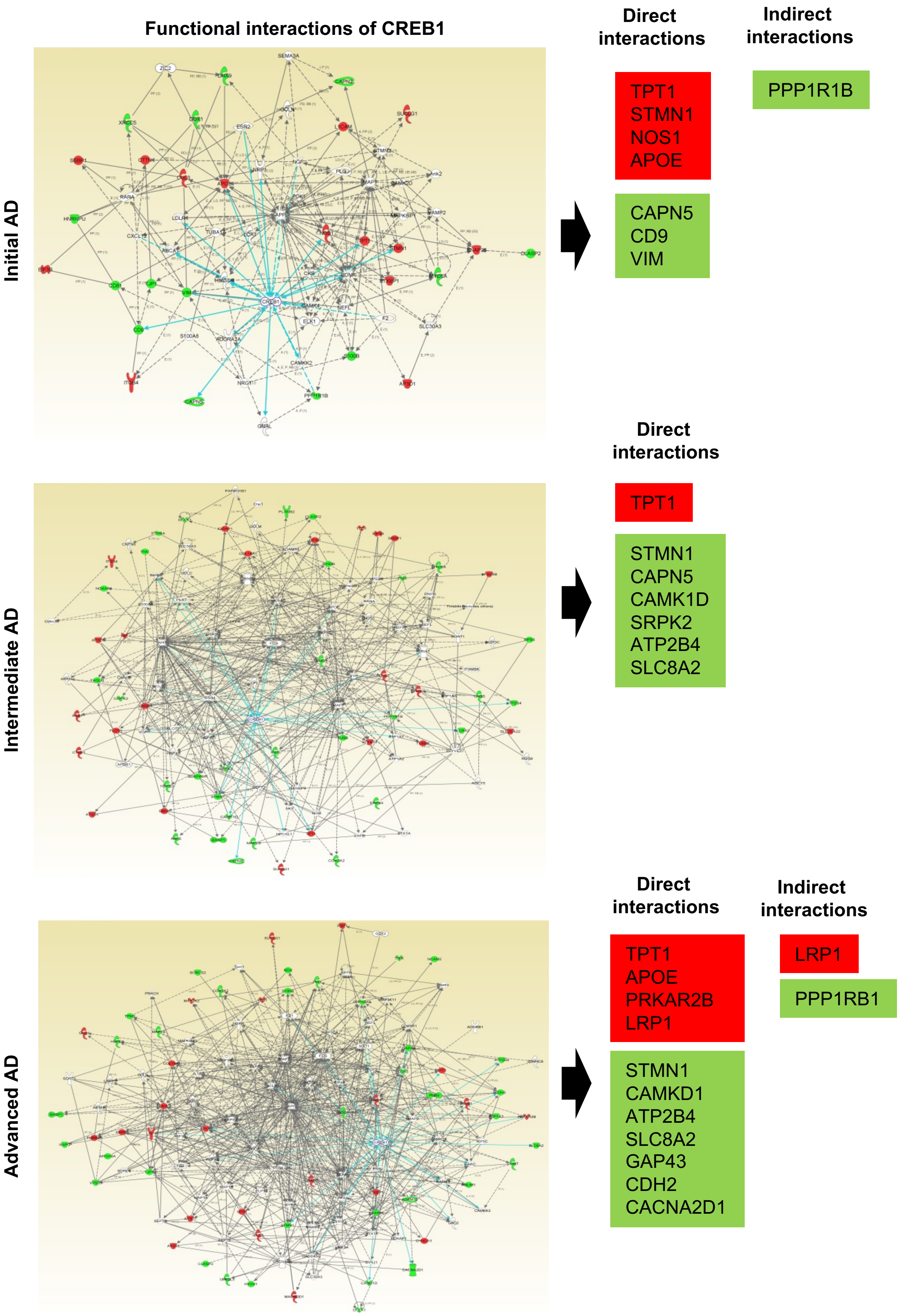

Supplementary figure 5. Modulation of CREB1 protein interaction network during the neurodegenerative process in the OB. For each AD stage, the CREB1 interactome is shown, where protein connections are indicated with blue lines (left panels), highlighting indirect and direct targets (right).

Intermediate AD

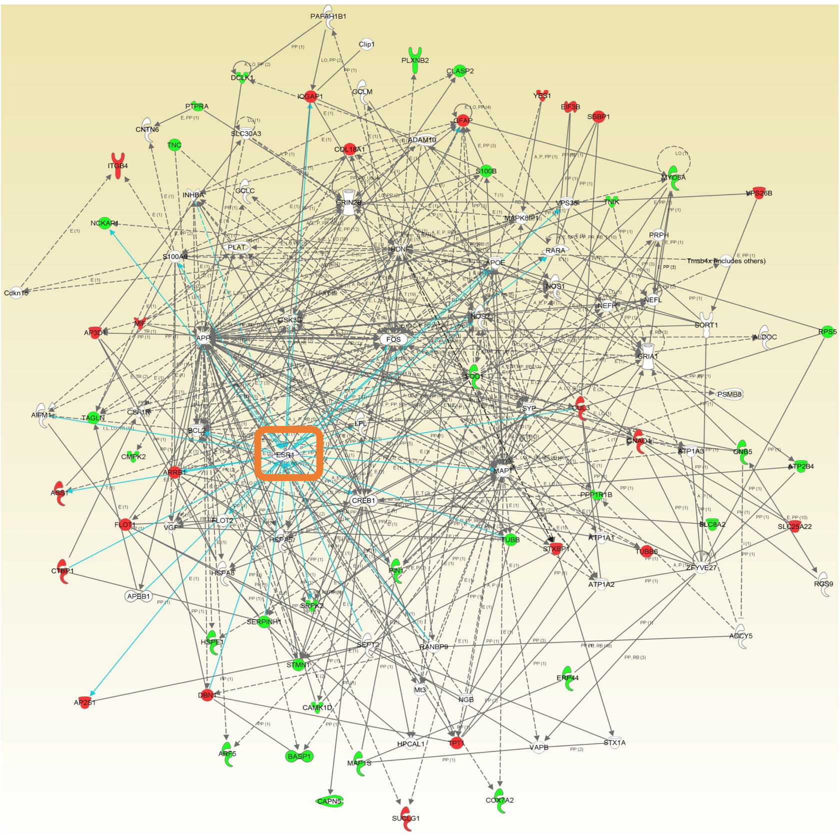

Advanced AD

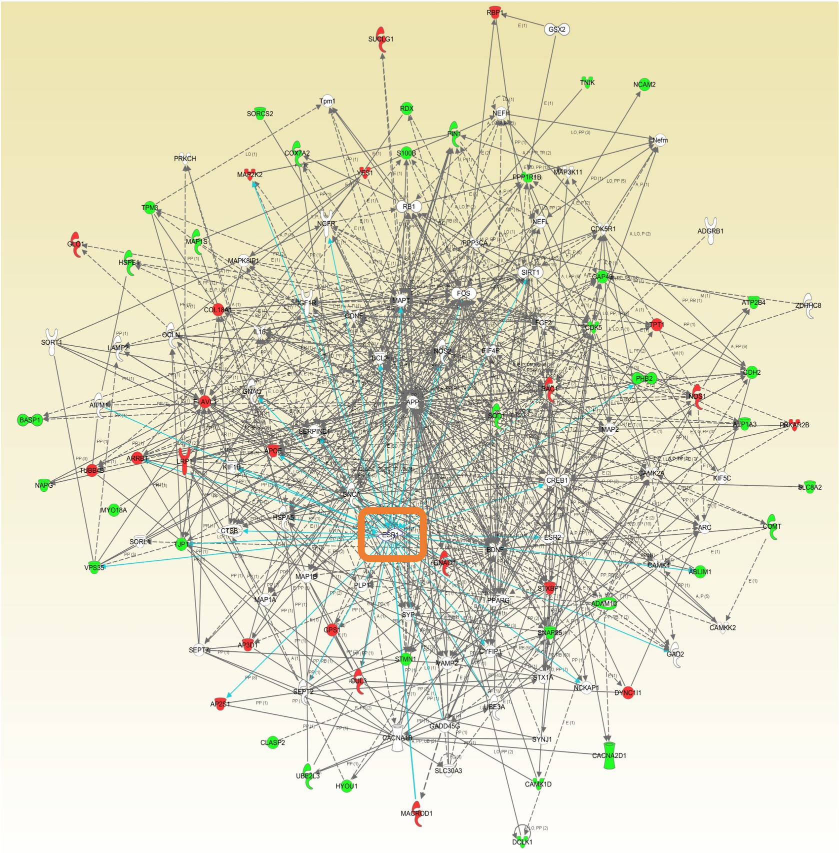

Supplementary Figure 6: Modulation of the olfactory bulb ESR1 (left, blue lines) interactome during AD progression

- Supplementary Information -

Antibody quality controls, loading controls,
And full-length gels and blots

Antibody quality control

Quality controls have been performed for antibodies used in this study. To monitor the specificity, Western-blotting of whole protein extracts derived from tissues & cell lines (human and mouse origin) were performed:

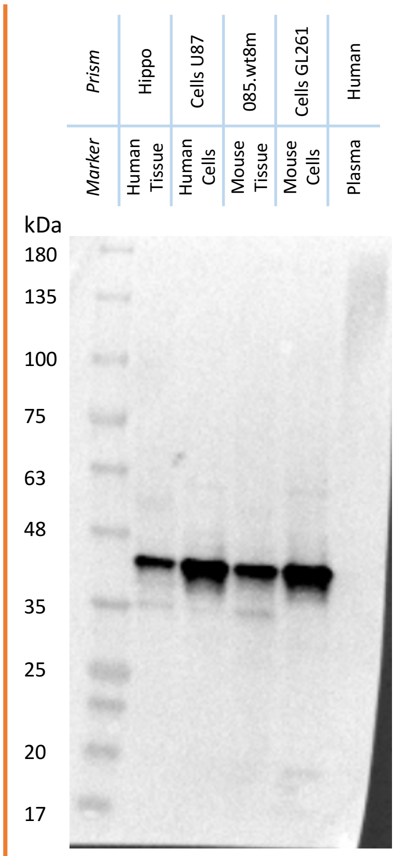

P38-43kDa

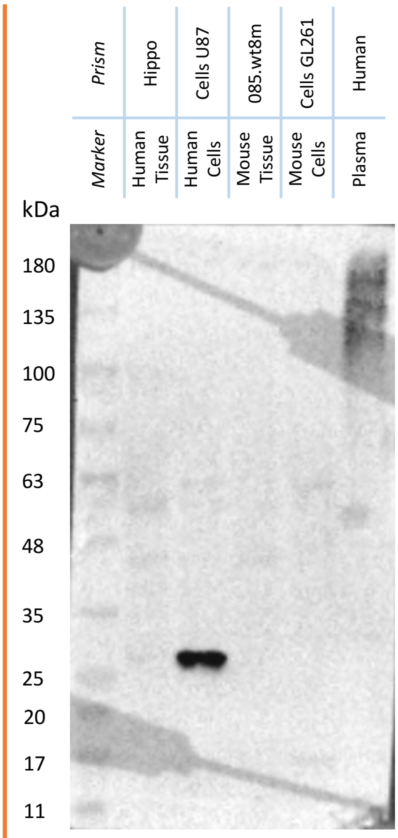

pHSP27-27kDa

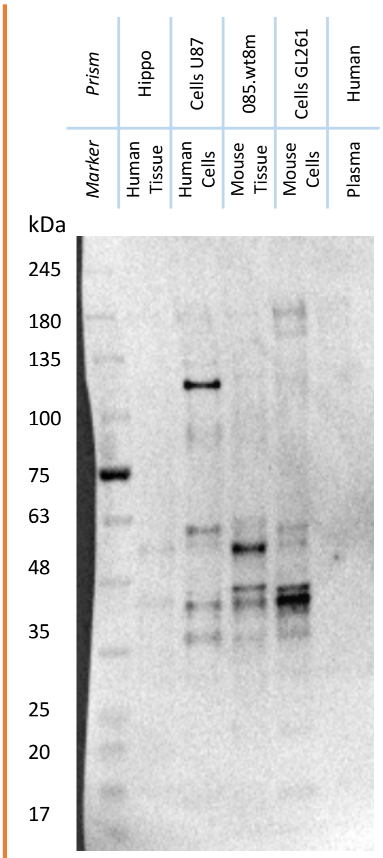

pP38-43kDa

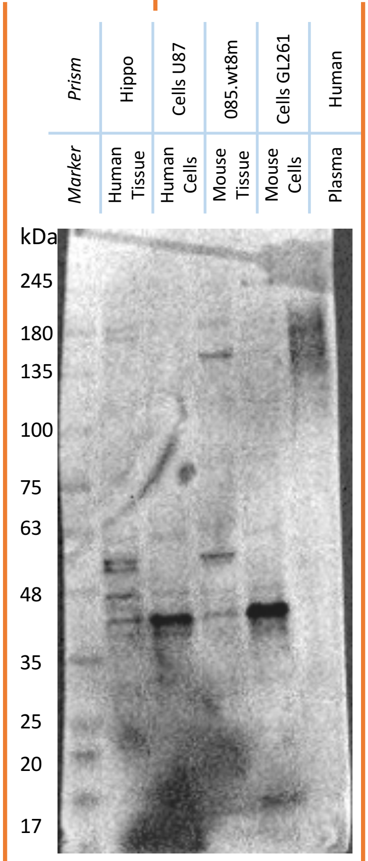

MKK3-40kDa

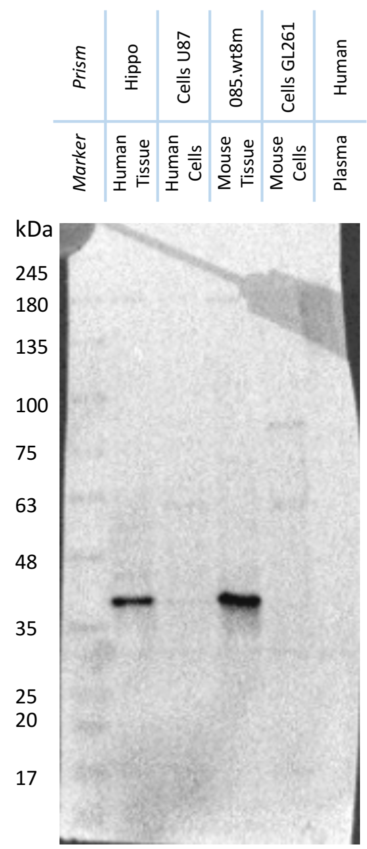

MKK6-38kDa

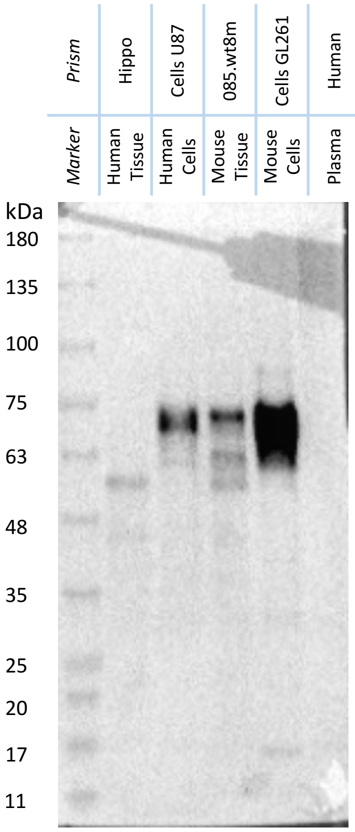

pATF2-70kDa

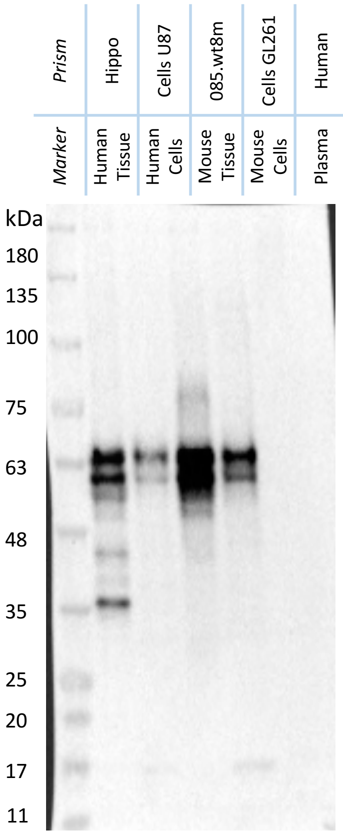

pPDK1-58-68kDa

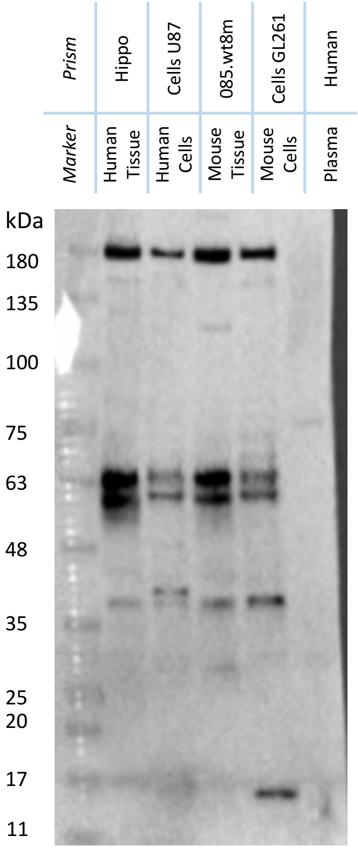

PDK1-58-68kDa

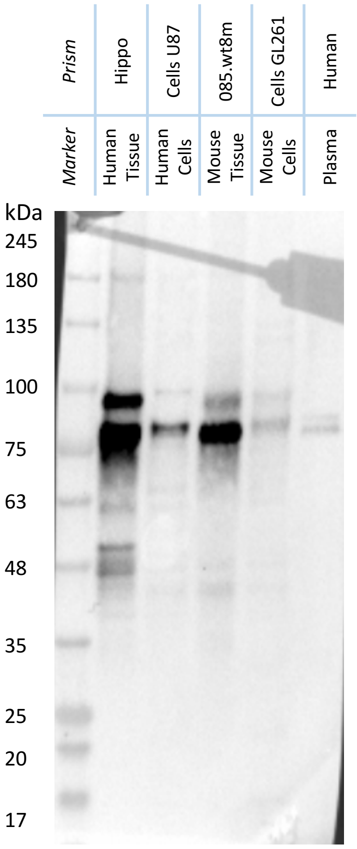

pPKC pan-78-82-82-85kDa

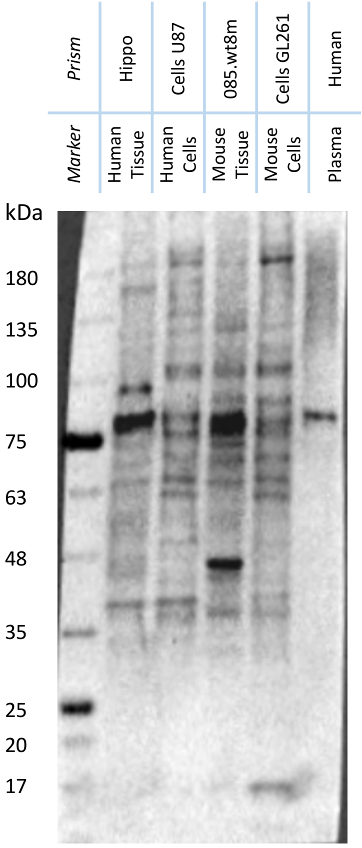

PKC pan-78-82-82-85kDa

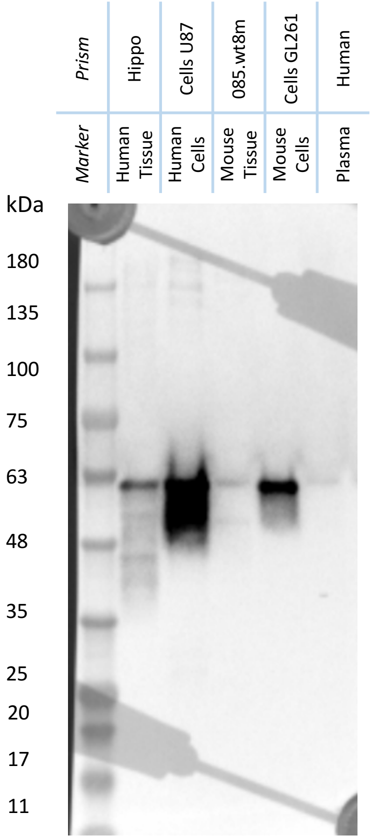

Vimentin-50kDa

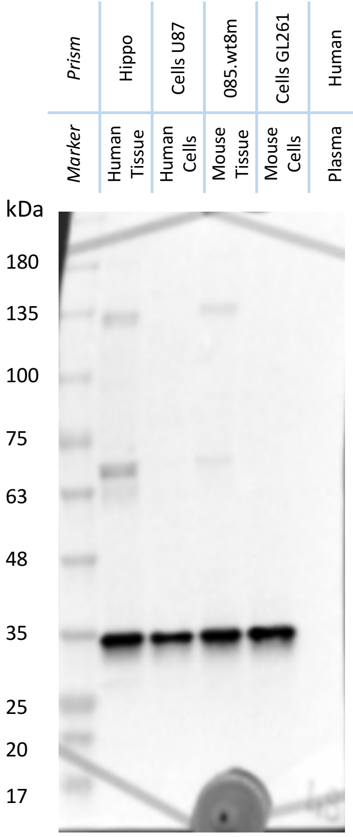

PHB2-33kDa

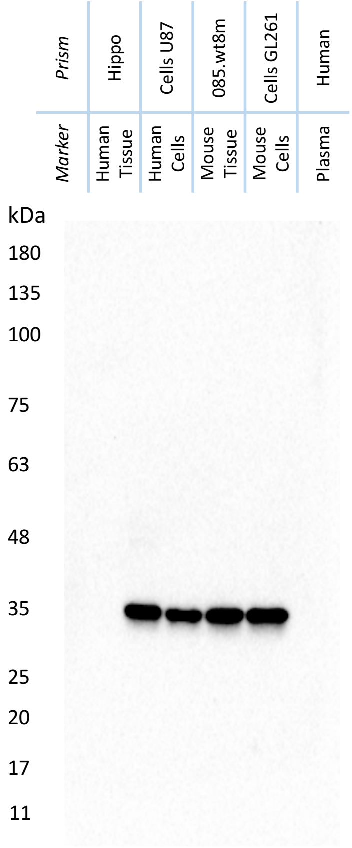

PHB1-32kDa

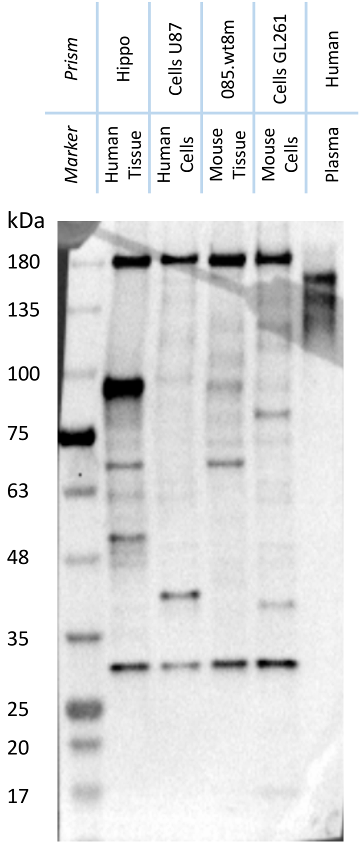

pPHB1 T258-32kDa

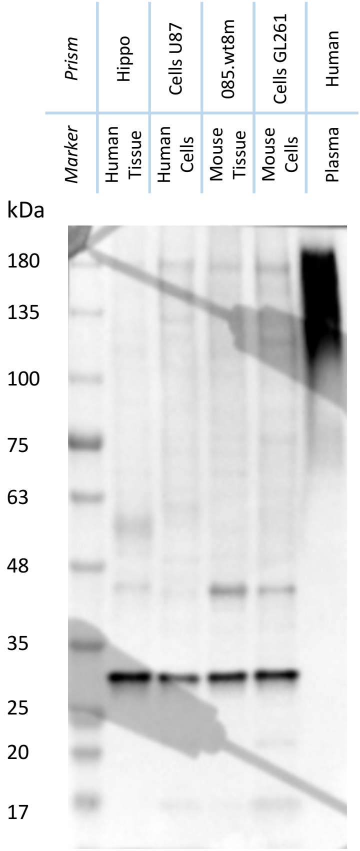

pPHB1 Y259-32kDa

Western blot images were taken using the instrument Chemidoc MB Imaging System, from BioRad. Densitometric analysis was performed using Image Lab 5.2 software. The instrument settings were optimized for each antibody. The application with predefined image acquisition settings optimal for our sample was: *Blot and High Resolution*. The specific settings for the imaging area depended on the gel type used in each case: *''BioRad Criterion Gel'' or ''BioRad MiniProtean Gel''.* In the Image exposure setting different modes were chosen either for faint or for intense bands. Automatic exposure mode was selected for those antibodies with good signal, in which the software automatically optimizes the exposure time for the best picture. Manual exposure mode was used for antibodies less sensitive, in which the user decides the best exposure time for each picture.

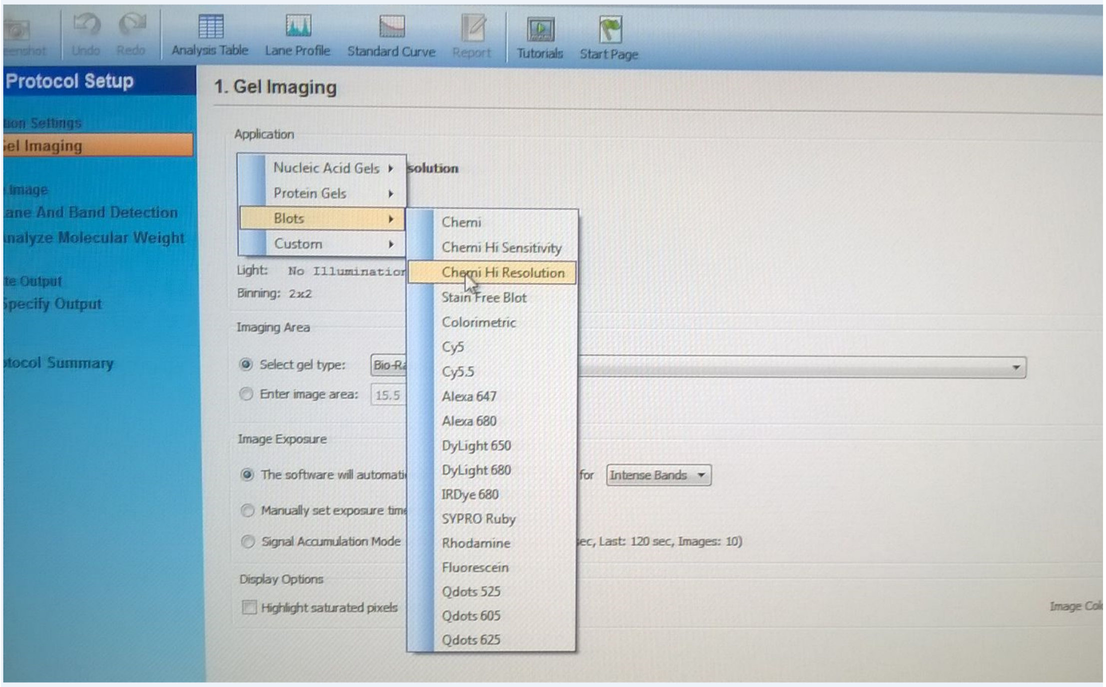

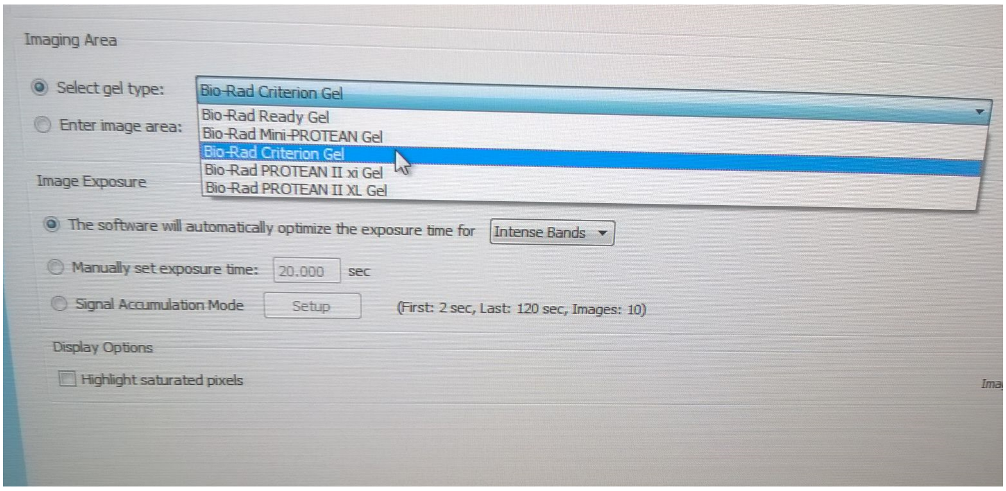

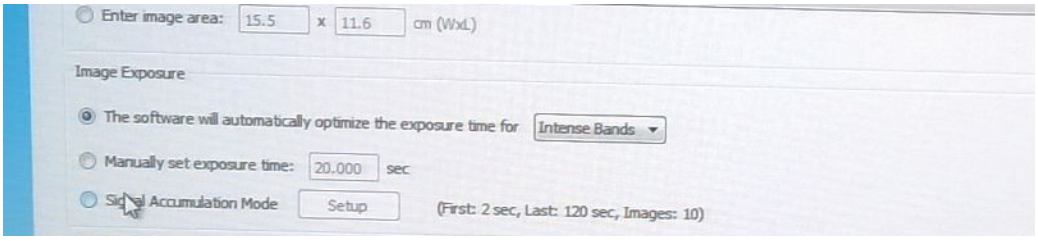

Loading controls, and full-length gels/blots corresponding to figure 4a

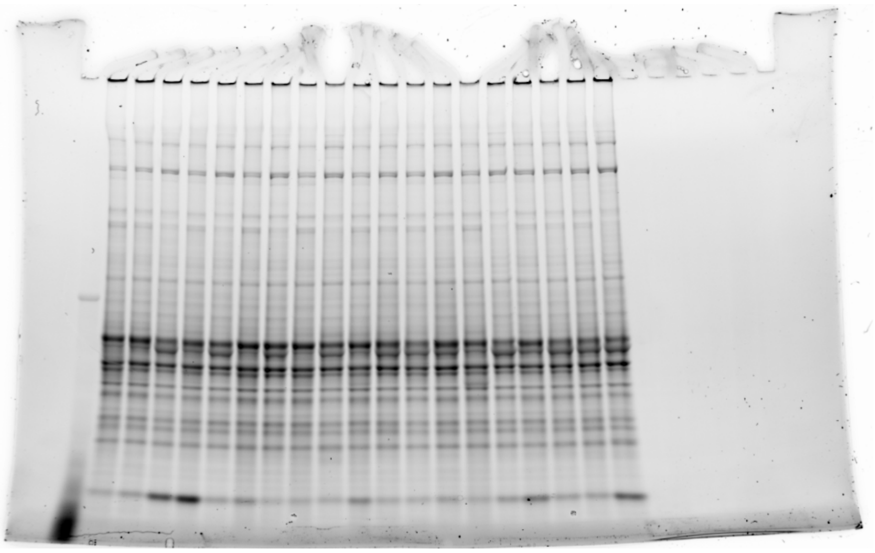

Gel stain-free
Acquisition time: 2.5min

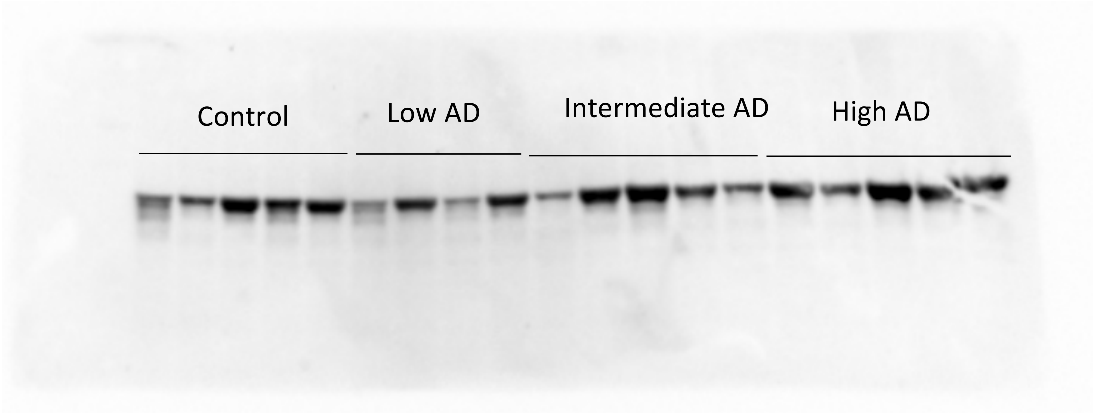

Antibody:pMKK3/6
Exposure time: 60s
Acquisition mode: manual

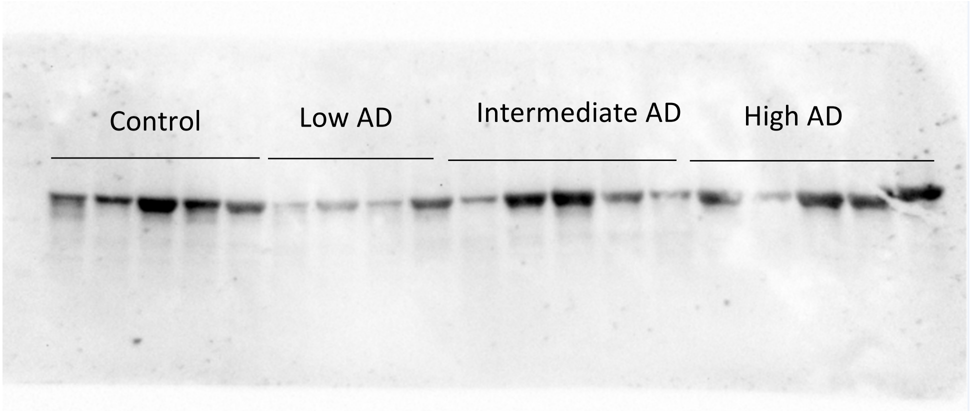

Antibody:MKK3
Exposure time: 100s
Acquisition mode: manual

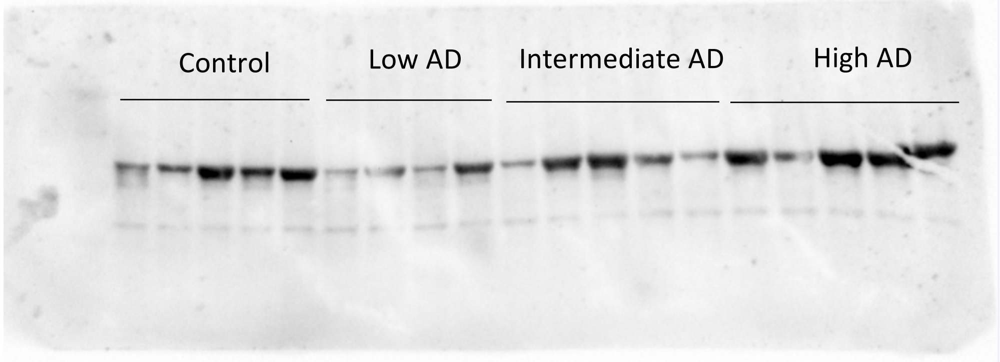

Antibody:MKK6
Exposure time: 100s
Acquisition mode: manual

Loading controls, and full-length gels/blots corresponding to figure 4b

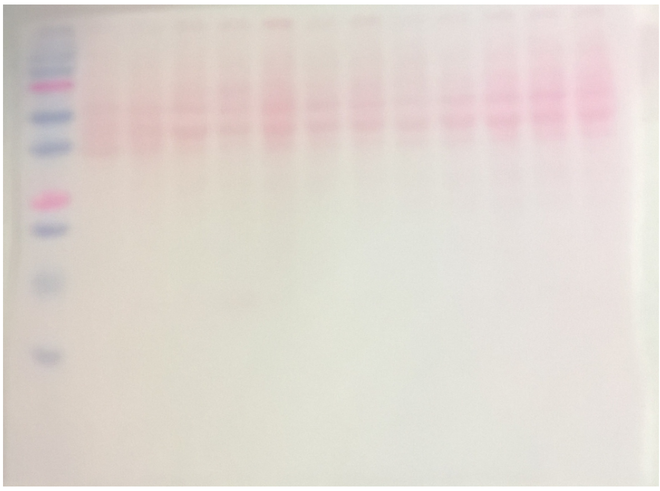

Blot. Ponceau staining

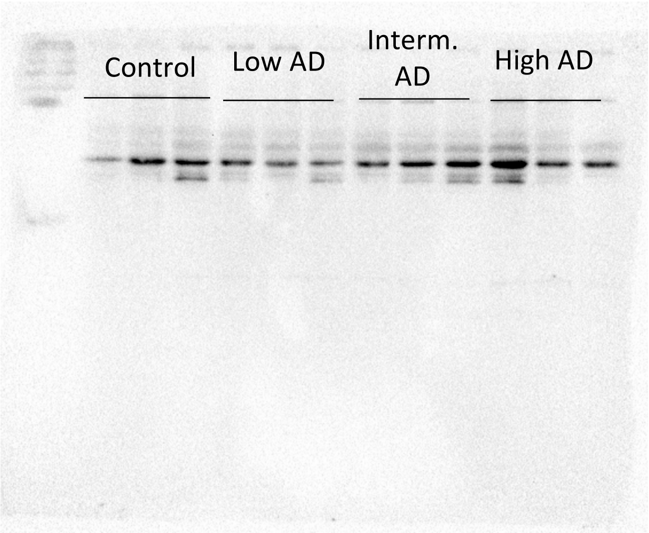

Antibody:p-p38
Exposure time: 15s
Acquisition mode: auto

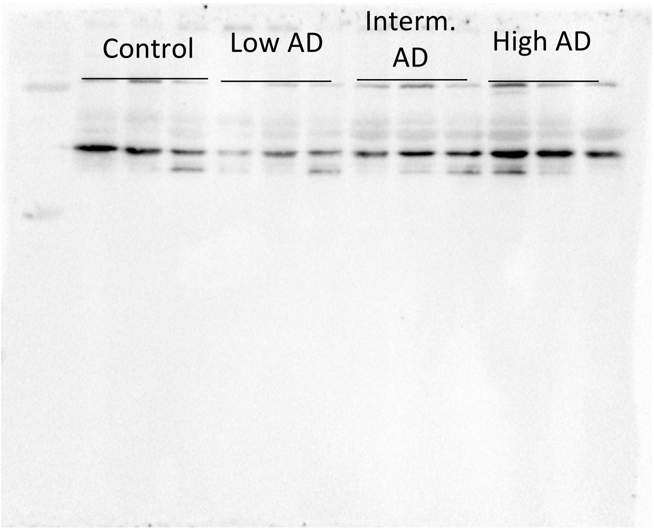

Antibody: p38
Exposure time: 41s
Acquisition mode: auto

Loading controls, and full-length gels/blots corresponding to figure 4b

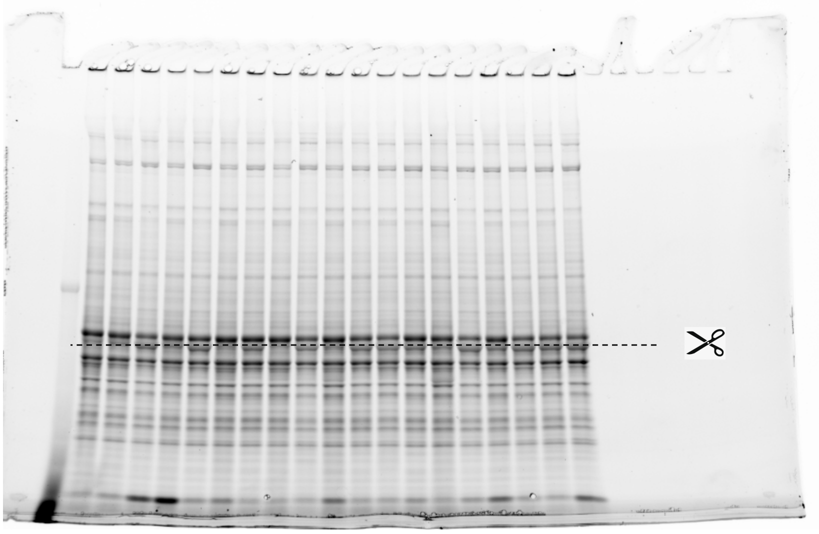

Gel stain-free
Acquisition time: 2.5min

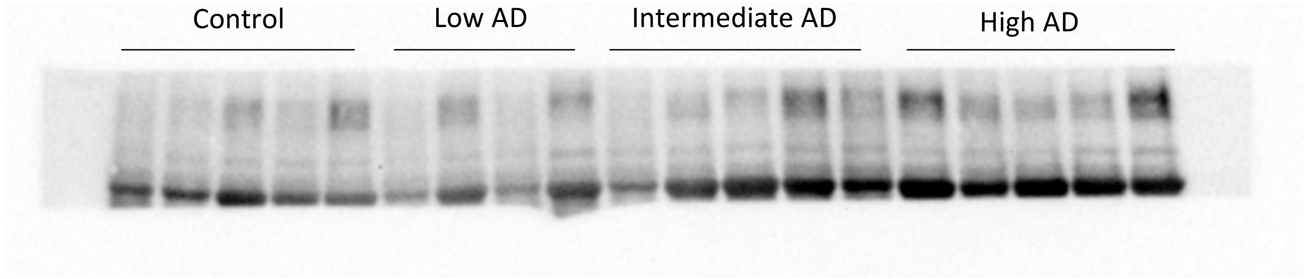

Antibody:p-ATF2; Exposure time: 100s; Acquisition mode: manual

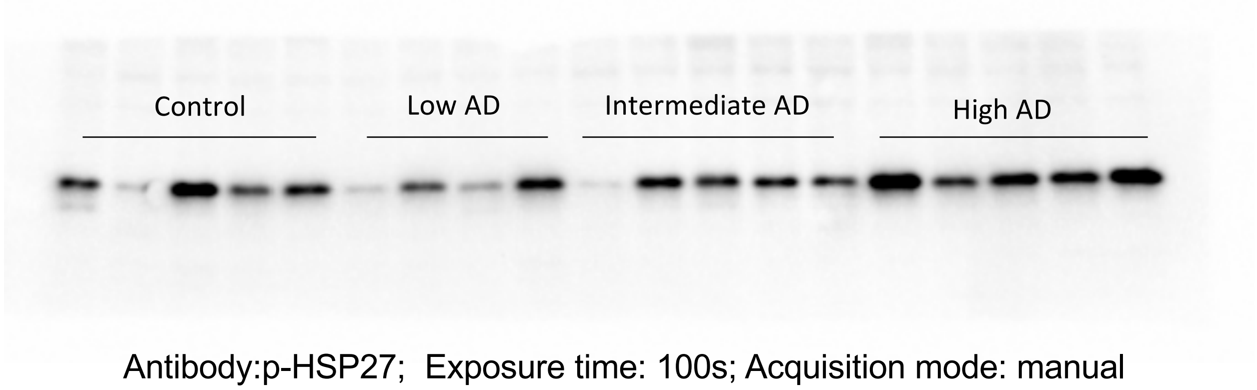

Antibody:p-HSP27; Exposure time: 100s; Acquisition mode: manual

Loading controls, and full-length gels/blots corresponding to figure 4c

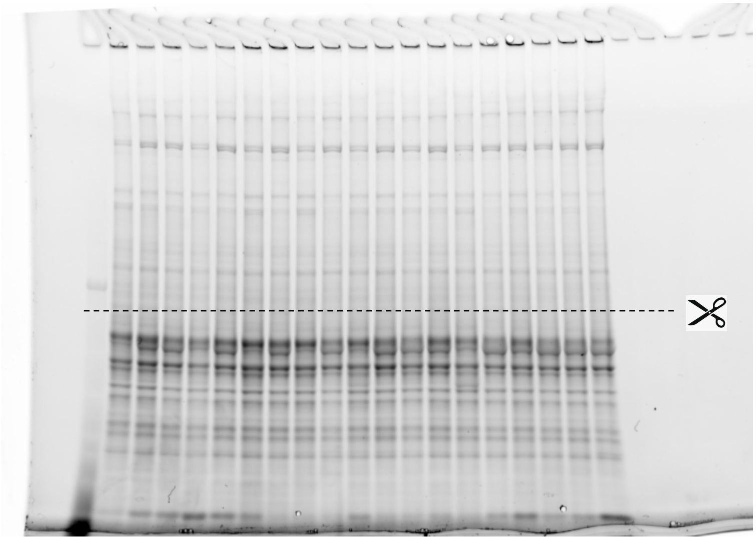

Gel stain-free
Acquisition time: 2.5min

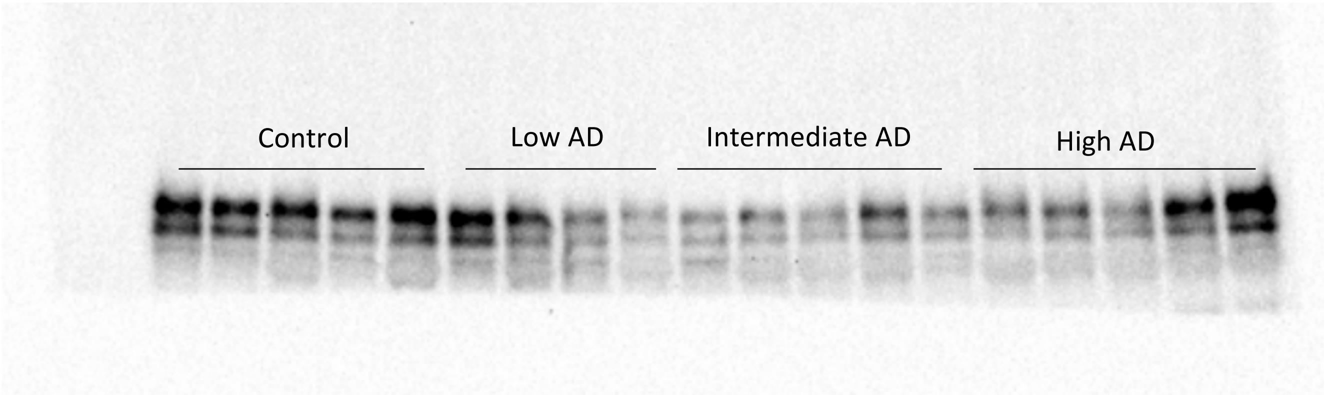

Antibody: p-PDK1; Exposure time: 100s; Acquisition mode: manual

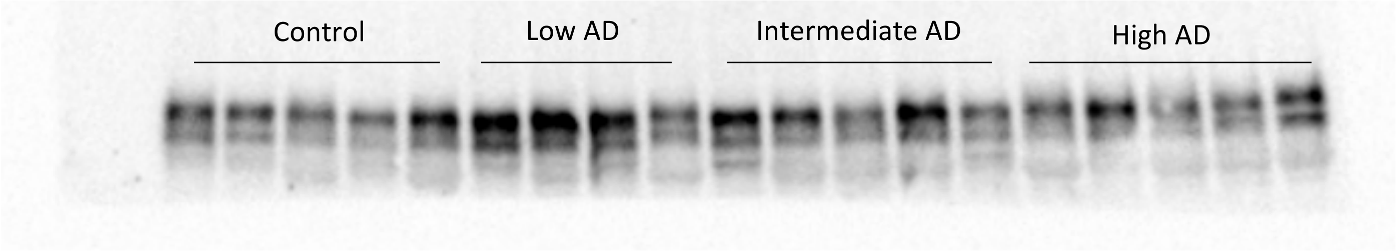

Antibody: PDK1; Exposure time: 100s; Acquisition mode: manual

Loading controls, and full-length gels/blots corresponding to figure 4c

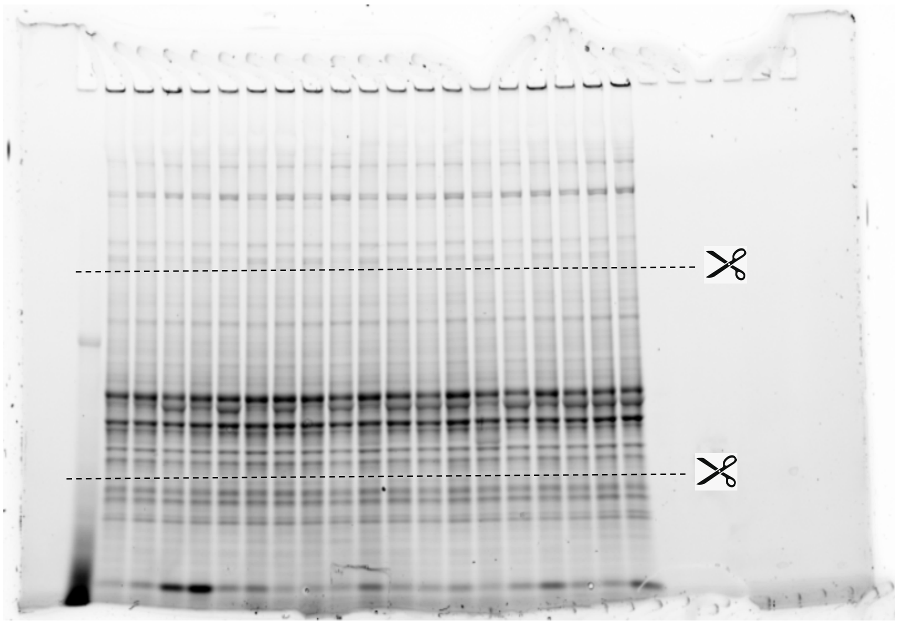

Gel stain-free
Acquisition time: 2.5min

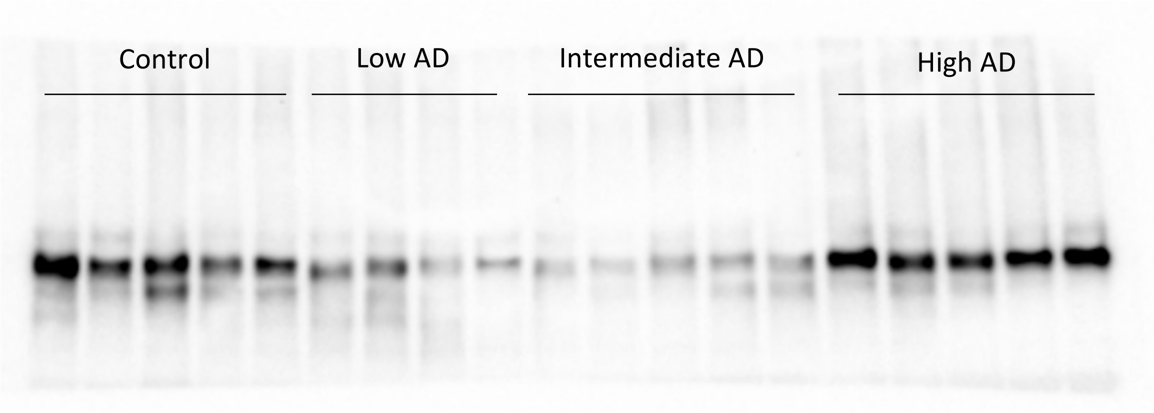

Antibody: p-PKC-Pan; Exposure time: 100s; Acquisition mode: manual

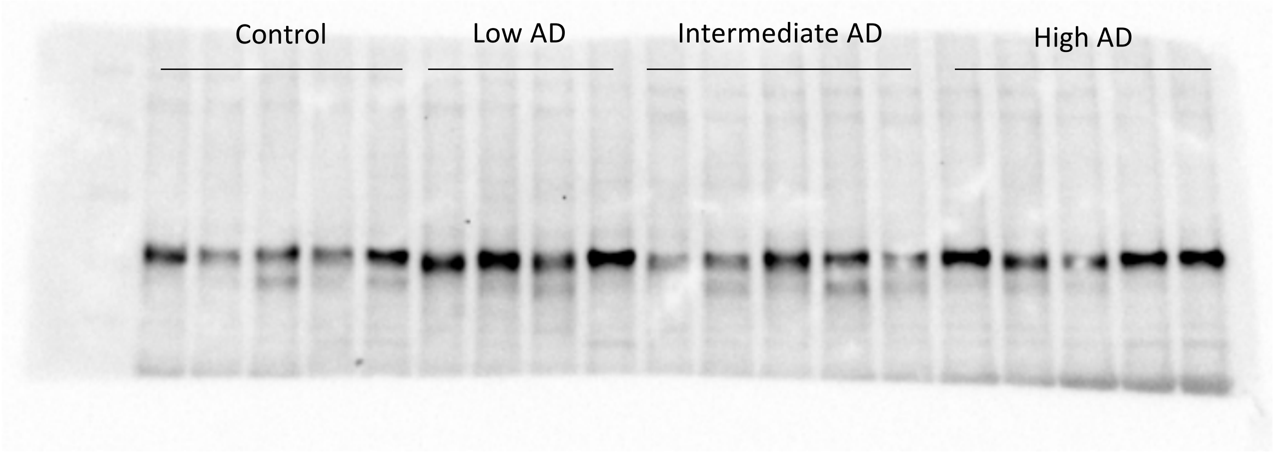

Antibody: PKC-Pan; Exposure time: 100s; Acquisition mode: manual

Loading controls, and full-length gels/blots corresponding to figure 7a

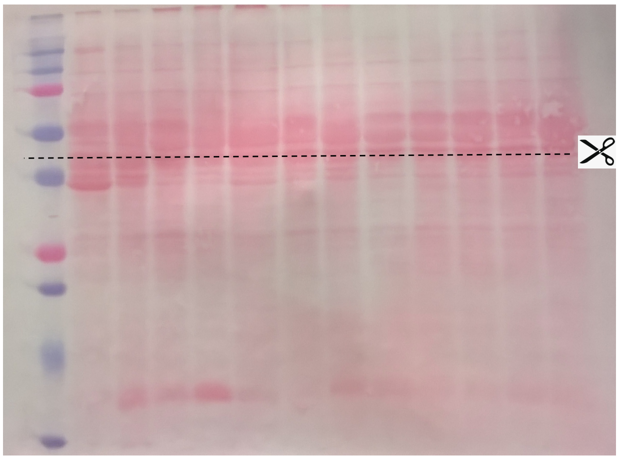

Blot. Ponceau staining

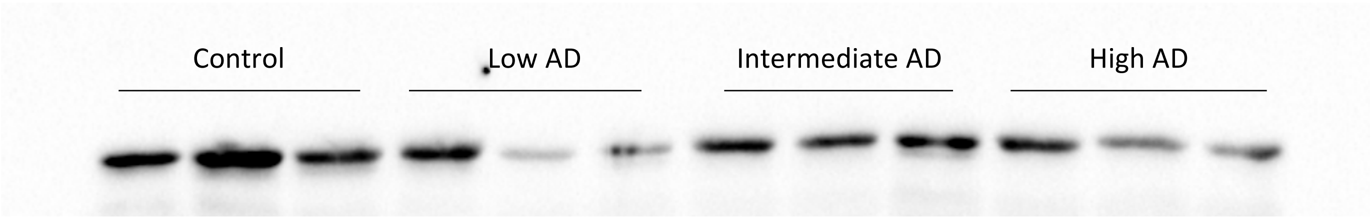

Antibody: Vimentin; Exposure time: 55s; Acquisition mode: manual

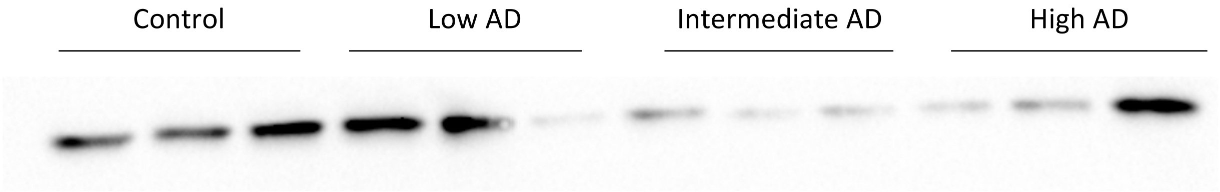

Antibody: PHB2; Exposure time: 55s; Acquisition mode: manual

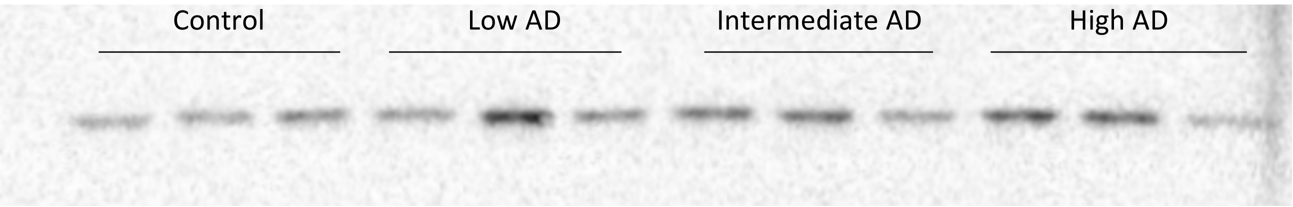

Antibody: PHB1; Exposure time: 120s; Acquisition mode: manual

Loading controls, and full-length gels/blots corresponding to figure 7b

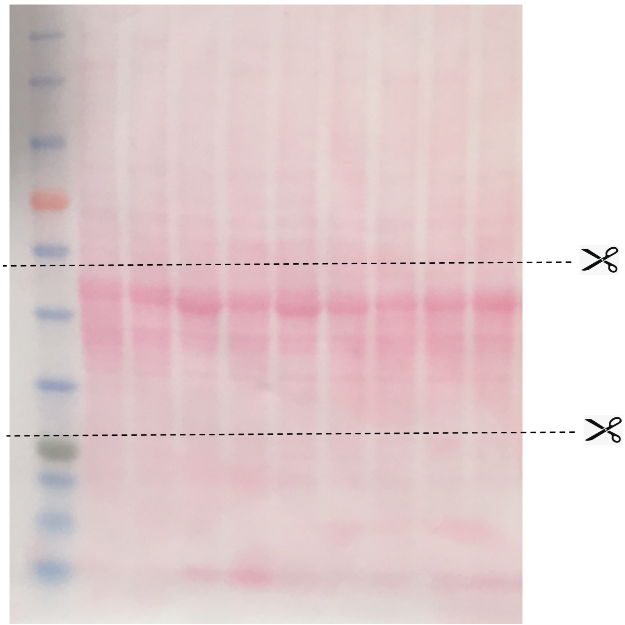

Blot. Ponceau staining

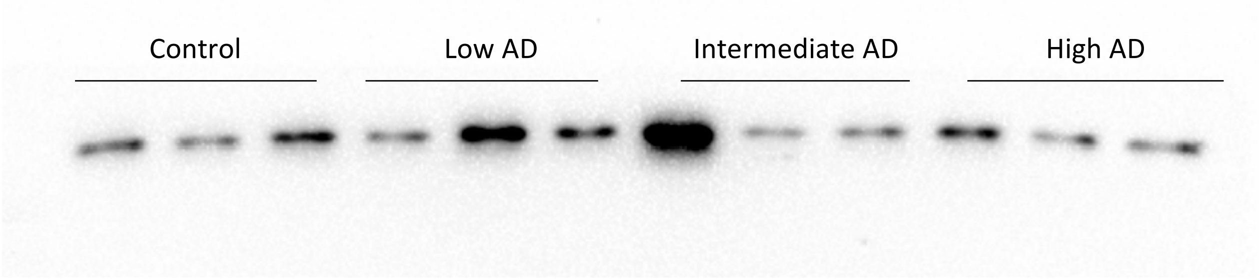

Antibody: p-PHB1 (258); Exposure time: 35.3s; Acquisition mode: auto

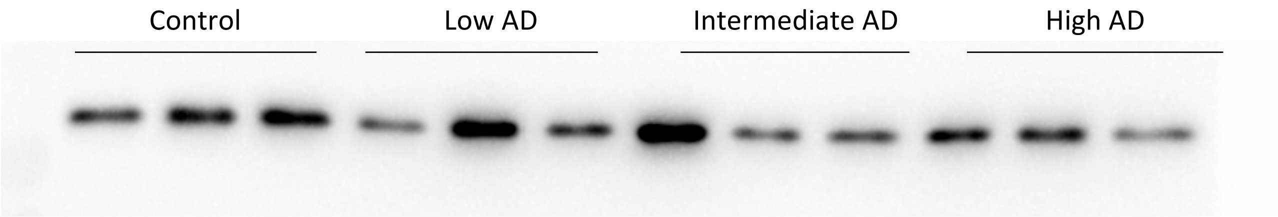

Antibody: p-PHB1 (259); Exposure time: 5.48s; Acquisition mode: auto

Loading controls, and full-length gels/blots corresponding to figure 8a

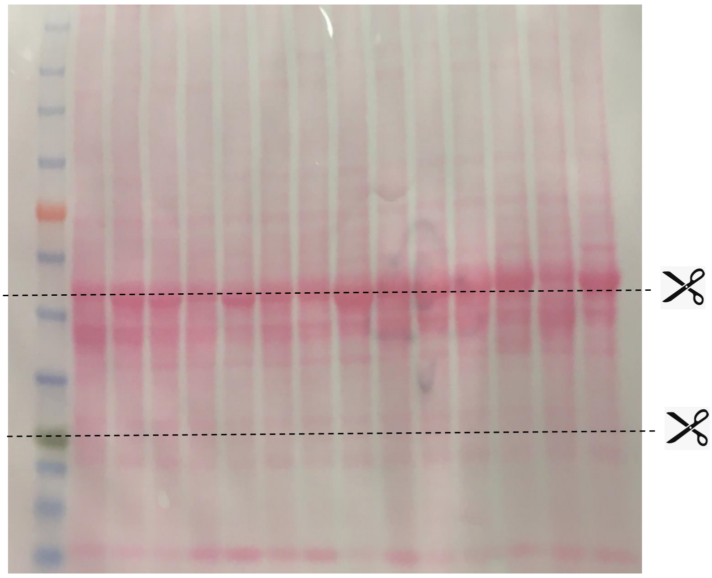

Blot. Ponceau staining

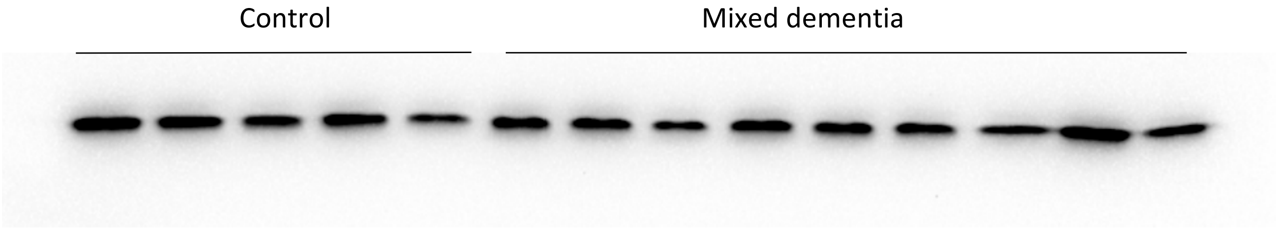

Antibody: PHB1; Exposure time: 28.6s; Acquisition mode: auto

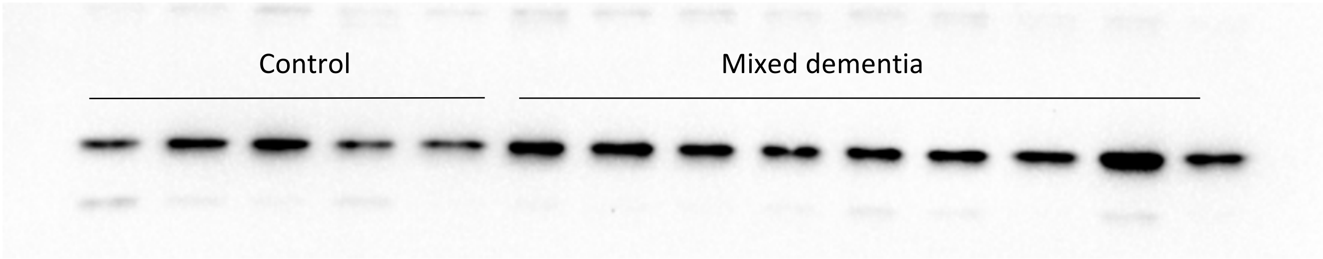

Antibody: PHB2; Exposure time: 40.3s; Acquisition mode: auto

Loading controls, and full-length gels/blots corresponding to figure 8a

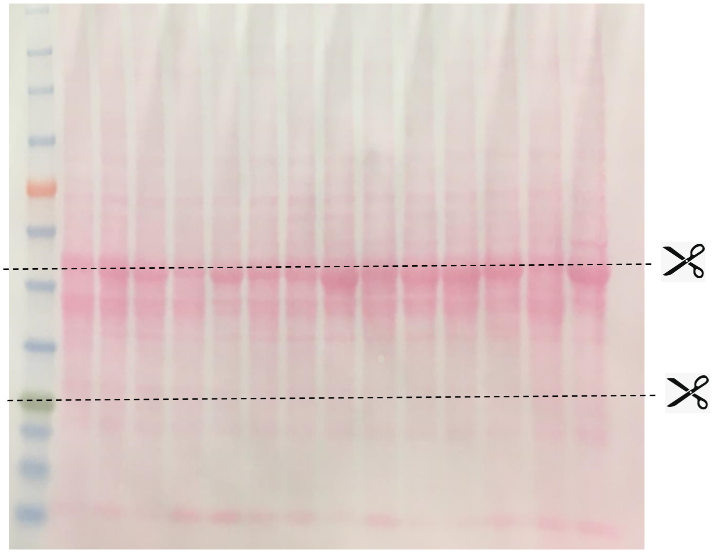

Blot. Ponceau staining

Control Mixed dementia

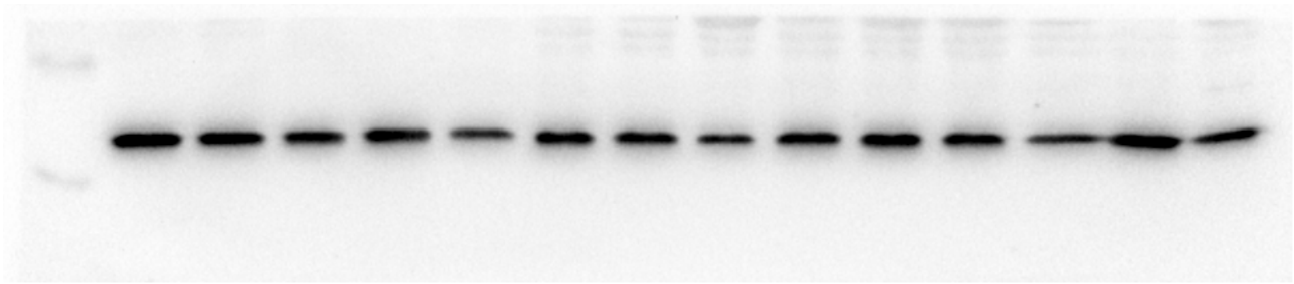

Antibody: p-PHB1 (258); Exposure time: 120s; Acquisition mode: manual

Control Mixed dementia

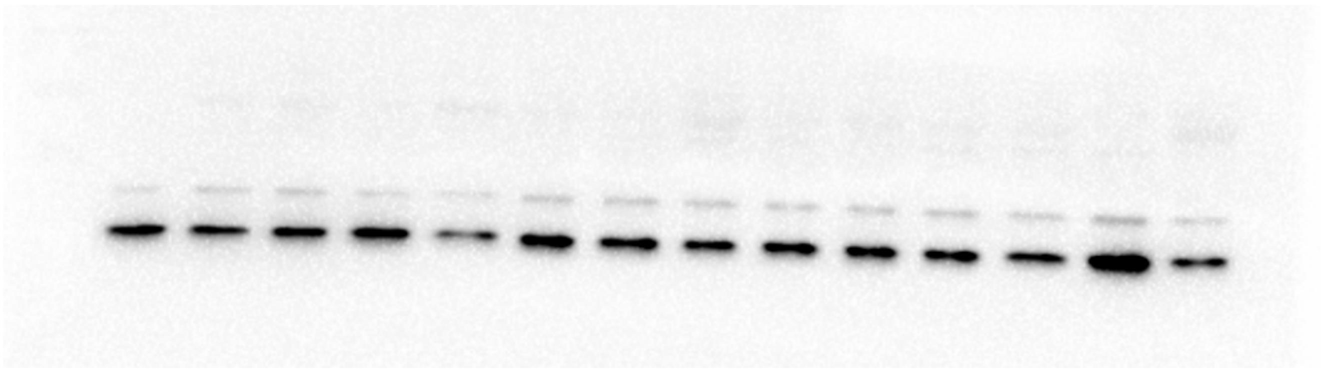

Antibody: p-PHB1 (259); Exposure time: 6.8s; Acquisition mode: auto

Loading controls, and full-length gels/blots corresponding to figure 8b

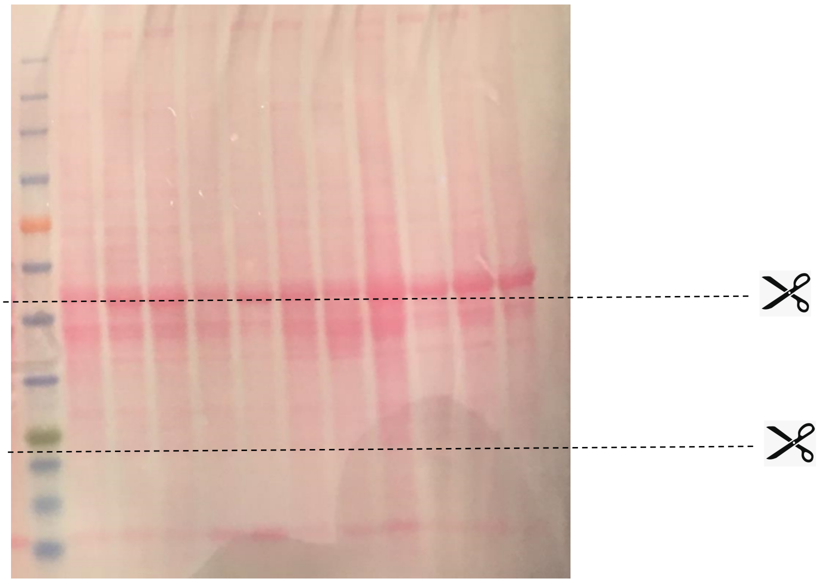

Blot. Ponceau staining

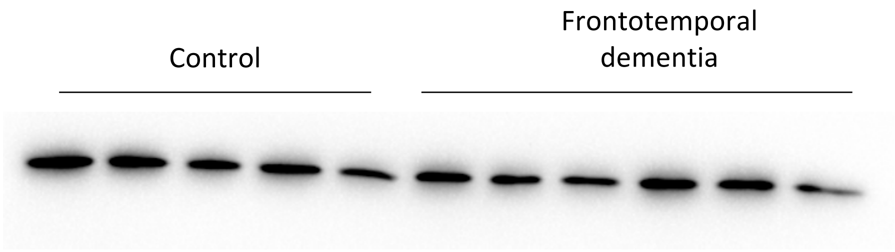

Antibody: PHB1; Exposure time: 28.6s; Acquisition mode: auto

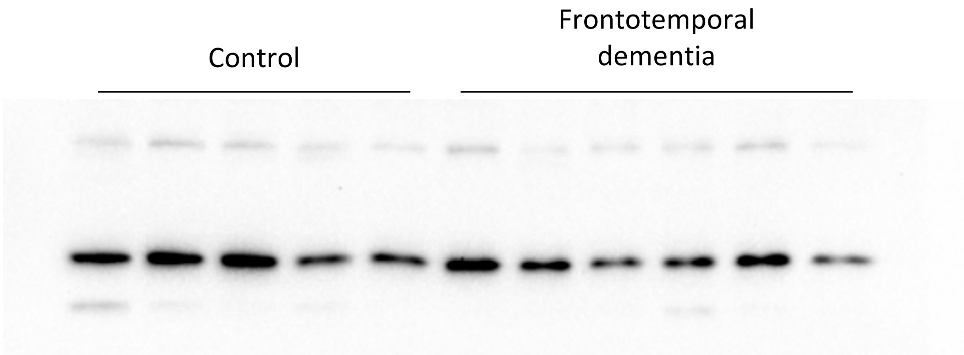

Antibody: PHB2; Exposure time: 40.3s; Acquisition mode: auto

Loading controls, and full-length gels/blots corresponding to figure 8b

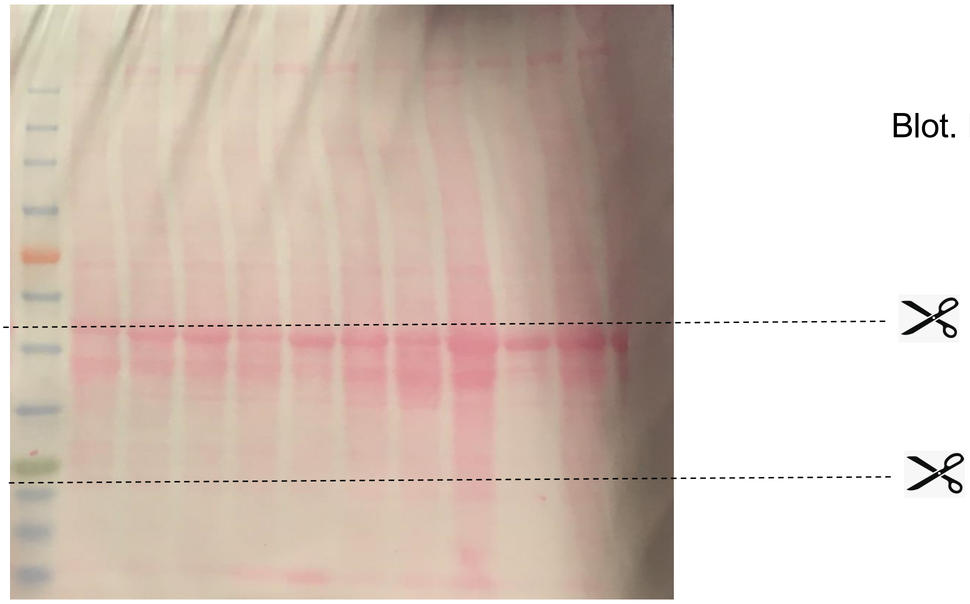

Blot. Ponceau staining

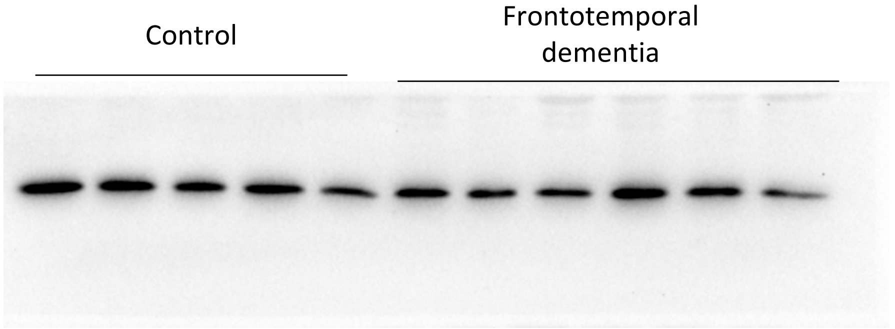

Antibody: p-PHB1 (258); Exposure time: 120s; Acquisition mode: manual

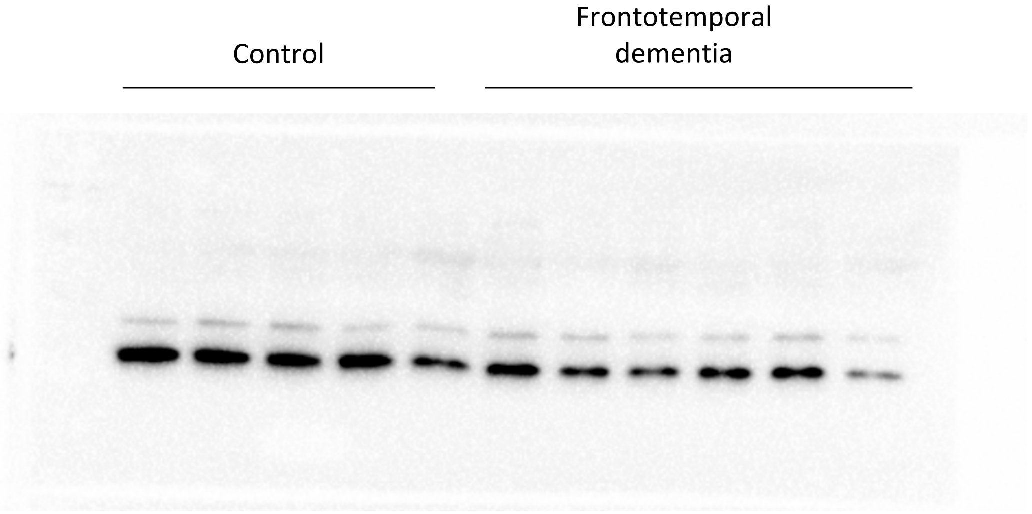

Antibody: p-PHB1 (259); Exposure time: 6.8s; Acquisition mode: auto

Loading controls, and full-length gels/blots corresponding to figure 8c

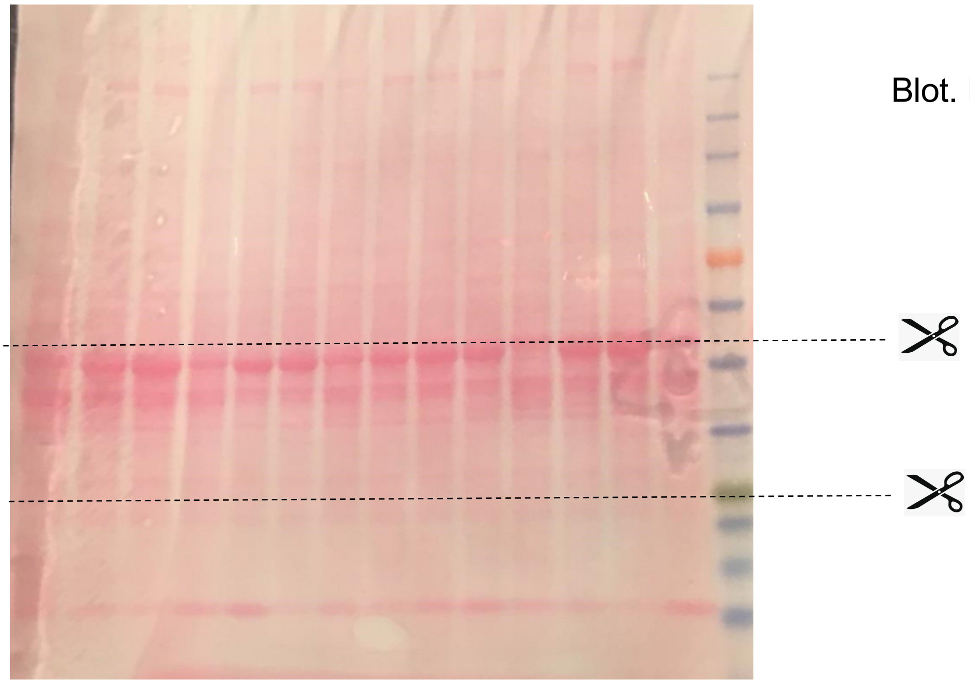

Blot. Ponceau staining

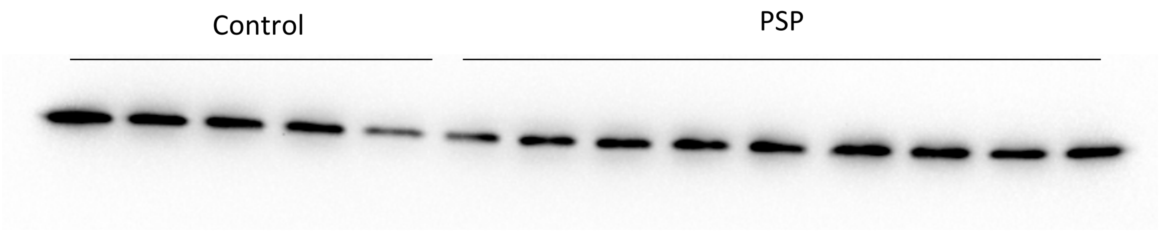

Antibody: PHB1; Exposure time: 28.6s; Acquisition mode: auto

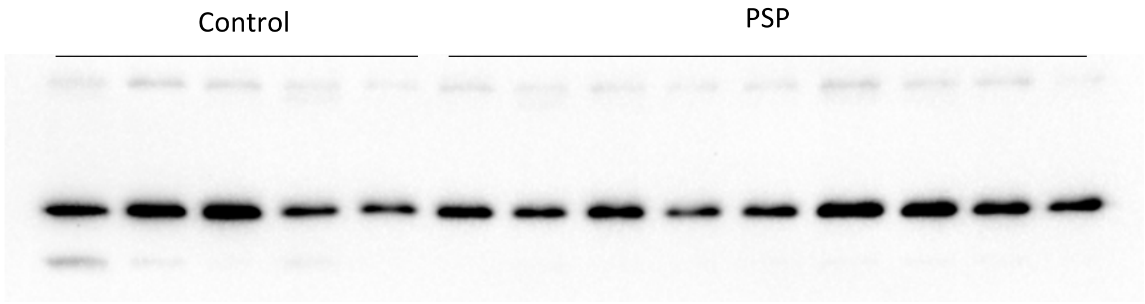

Antibody: PHB2; Exposure time: 40.3s; Acquisition mode: auto

Loading controls, and full-length gels/blots corresponding to figure 8c

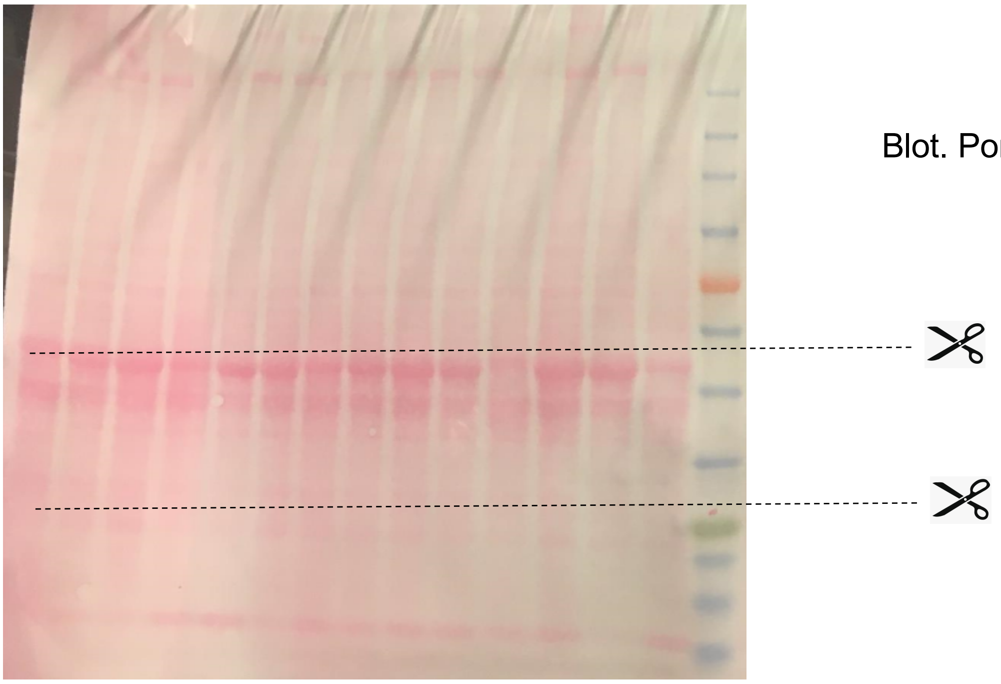

Blot. Ponceau staining

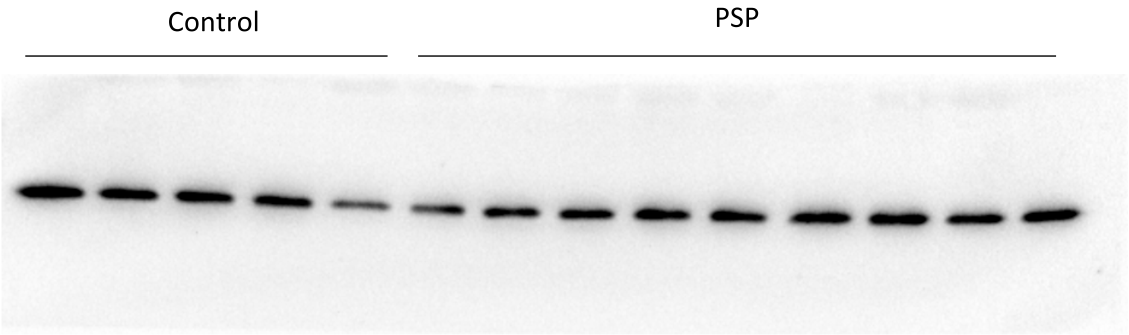

Antibody: p-PHB1 (258); Exposure time: 120s; Acquisition mode: manual

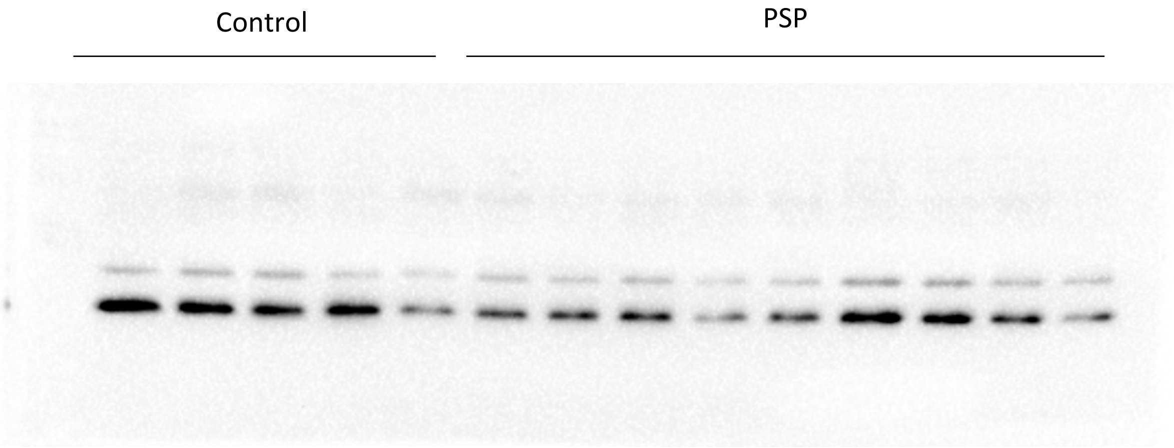

Antibody: p-PHB1 (259); Exposure time: 6.8s; Acquisition mode: auto
